# Disparate impacts on online information access during the Covid-19 pandemic

**DOI:** 10.1038/s41467-022-34592-z

**Published:** 2022-11-19

**Authors:** Jina Suh, Eric Horvitz, Ryen W. White, Tim Althoff

**Affiliations:** 1 Microsoft Research, Redmond, WA USA; 2 University of Washington, Seattle, WA USA

**Keywords:** Risk factors, Society

## Abstract

The COVID-19 pandemic has stimulated important changes in online information access as digital engagement became necessary to meet the demand for health, economic, and educational resources. Our analysis of 55 billion everyday web search interactions during the pandemic across 25,150 US ZIP codes reveals that the extent to which different communities of internet users enlist digital resources varies based on socioeconomic and environmental factors. For example, we find that ZIP codes with lower income intensified their access to health information to a smaller extent than ZIP codes with higher income. We show that ZIP codes with higher proportions of Black or Hispanic residents intensified their access to unemployment resources to a greater extent, while revealing patterns of unemployment site visits unseen by the claims data. Such differences frame important questions on the relationship between differential information search behaviors and the downstream real-world implications on more and less advantaged populations.

## Abstract (web summary)

The COVID-19 pandemic has stimulated an important changes in online information access. Here, the authors analyse everyday web search interactions across 25,150 US ZIP codes revealing significant differences in how digital informational resources are mobilized by different communities.

## Introduction

Socioeconomic and environmental factors play a significant role in the health and well-being of individuals and communities^1–3^. Despite pandemic-driven efforts to close the long-term and emergent health equity gap^2^, studies during the COVID-19 pandemic have demonstrated that socioeconomically and environmentally disadvantaged subpopulations have been disproportionately and negatively affected by the disease^4–6^, with threefold higher infection rates and sixfold higher death rates in predominantly Black US counties than in white counties^7^. In recent decades, digital access has also gained attention as an important factor modulating health outcomes, as individuals harness the internet to seek health information and to access healthcare services (i.e., telehealth, online pharmacy)^8^. During the COVID-19 pandemic, digital engagement in resources across health, educational, economic, and social needs grew in importance because of lockdown mandates, social isolation, and economic burdens^9–11^ as well as due to internet-based communication methods employed by public institutions, such as the online dissemination of COVID-related information by the World Health Organization^10^.

Unfortunately, disparities in digital access also reflect socioeconomic and environmental dimensions of variation^12^. The most basic form of digital inequality, the so-called first-level digital divide, manifests itself as the difference between adequate and inadequate digital infrastructure and devices (i.e., access to technology or the quality of access)^13^. Digital inequalities also manifest themselves as the differences in the usage of digital technologies and skills relevant to the usage of digital technologies, the so-called second-level digital divide^14,15^.

In this study, we harness the centrality of web search engines for online information access to observe the second-level digital divide at population scales. We conduct a retrospective and longitudinal observational study using search data to quantify the changes during the pandemic in how offline exclusion (e.g., lack of sufficient economic resources, lack of health insurance) relates to changes to existing digital exclusion (e.g., reduced participation in online banking or eHealth).

This study extends prior work on pandemic-related disparities, many of which concern the epidemiological dynamics of the pandemic^4–7^. Leveraging web search interactions enables us to model users’ search interests which are reflective of their underlying resource needs^16–18^. This includes the use of critical digital resources such as online educational sites in response to school closures, online food delivery information in response to restaurant closures, online social interactions in response to physical distancing and travel restrictions, or online unemployment and economic assistance in response to economic instability during the pandemic. Given that the pandemic has impacted everyone’s web search behaviors across many different topic categories, however closely related to the pandemic itself^9,19^, our goal and key contribution is to identify differences across communities in their digital behavioral responses to the pandemic and to discover potential barriers and challenges in accessing critical resources on the web.

Prior work on understanding digital disparities has relied on costly surveys, interviews, or self-reports^20–22^ that require direct engagement with the study population in order to prompt a recounting of their past behaviors rather than passively observing their actual behaviors. Datasets from specific service providers (e.g., Wikipedia^23^, Zearn.org^24,25^), domains (e.g., telehealth^26^, eHealth^27^), or geographic areas (e.g., Northern California^27^) do not capture digital behaviors across a broad spectrum of human needs and subpopulations and at fine geo-temporal granularities. Macroeconomic measures, such as unemployment claims, do not capture potentially unmet needs or access barriers (e.g., confusion around unemployment benefits^28–30^).

Conversely, web search logs are routinely collected on a near real-time basis and at large scales, providing unique opportunities to examine digital behaviors across a wide range of topics, geographies, and subpopulations as well as highlighting potential barriers and changes to such engagement behaviors^31^. In fact, web search logs have enabled studies of human behaviors across many different domains^32–35^, times^36–39^, locations^40,41^, and to make inferences about the future or to identify risk factors^19,42–45^. In the context of the COVID-19 pandemic, such data has stimulated a prolific range of research on physical^19,46^, psychological^47–49^, and socioeconomic^50,51^ well-being^9^. Therefore, our study also extends prior work on digital disparities research through near real-time, population-scale analysis across many different information domains to reveal naturalistic digital engagement patterns uniquely seen through search data.

The differential digital engagement patterns we present in this study has real-world downstream implications. Most recently, the third-level digital divide has been conceptualized as the differential ability to translate the use of digital technologies into favorable outcomes, particularly leading to negative downstream outcomes in offline realms such as occupational pursuits, healthcare, and social networking^52,53^. For example, digital footprint gap in the usage of information and communication technologies (ICTs) has been shown to surface during childhood and the entire life course along offline axes of socioeconomic status (SES). As a result, they may wind up with smaller social networks and limited employment opportunities^54^. Furthermore, even after controlling for internet access, those from higher SES or higher digital literacy integrate digital resources into their lives and use the internet for more capital-enhancing activities that are likely to result in more upwards mobility in the offline world^15,53,55,56^. Just as the social, economic, cultural, and personal offline resources can affect engagement in the corresponding digital fields, digital exclusion and the lack of engagement in digital resources can lead to negative offline consequences^57^ across the range of downstream outcomes in the domains of health^8,27,54^, education^58^, and employment^59,60^. Therefore, it is important to observe digital behaviors across subpopulations and scrutinize the role of digital inequalities in our society. In addition, disadvantaged subpopulations are already at a higher risk of COVID-19 infection and mortality with heavier pandemic-induced socioeconomic burdens, such that it is critical to ensure that digital inequalities do not exacerbate the disparate impacts of the pandemic even further^10^. Therefore, our data and approach of quantifying differential usage of search across subpopulations can provide an important empirical lens into digital disparities research.

We contribute to this literature by analyzing 55 billion everyday web search interactions across multiple devices and 25,150 US ZIP codes during the COVID-19 pandemic. Our dataset includes anonymized search queries to the Bing search engine and subsequently clicked website URLs from those queries. In our work, instead of focusing narrowly on a single topic, we aim to examine a spectrum of broader information domains to capture a holistic view of the changes in digital engagement during the pandemic^61^. Therefore, we structure our analysis according to the five social determinants of health (SDoH) categories defined by the US Department of Health^62^, which have been widely used as a holistic framework to describe a wide range of socioeconomic and environmental factors that determine one’s health, well-being, and quality of life. Each search interaction is classified into the categories of health, education, economic assistance, and food access that cover a broad range of critical resource needs (Supplementary Table 4). We link the search interactions from each United States ZIP code to their respective per-ZIP code census variables that broadly cover five SDoH categories: (1) Healthcare Access and Quality (through health insurance coverage), (2) Education Access and Quality (through educational attainment level), (3) Social and Community Context (through proportions of population represented by different race/ethnicity), (4) Economic Stability (through income and unemployment rate), and (5) Neighborhood and Built Environment (through population density and internet access).

**Fig. 1:**
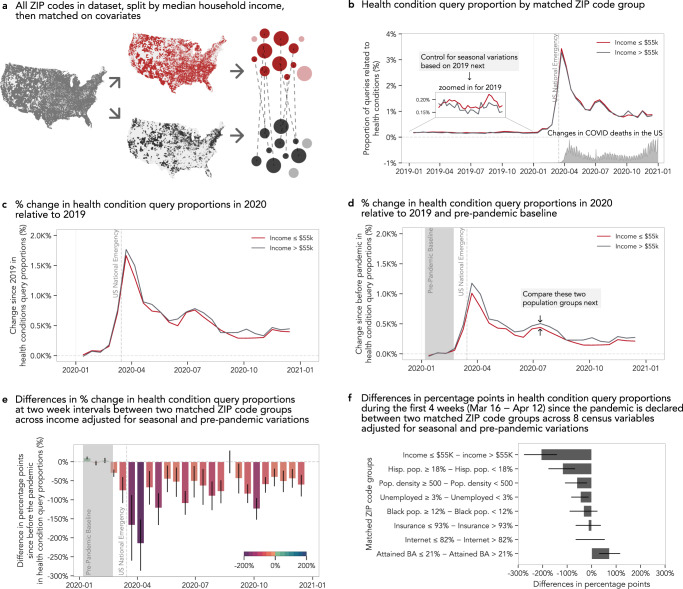
Quantifying disparities in online health information access. **a** 25,150 ZIP codes above and below *$*55,224 median household income are matched to control for other confounding covariates (see Methods). **b** The proportion of queries relating to a collection of health conditions in 2019 stay well below 0.25% of the total search queries across high-income (gray) and low-income (red) ZIP code groups, with mild seasonal highs around spring and fall and low-income group exhibiting slightly higher health condition query proportion. This proportion increases dramatically to over 3% around the time the US national emergency was declared and is elevated throughout 2020 as COVID death rates change over time. **c** Seasonal and weekly variations are accounted relative to 2019. **d** After accounting for pre-pandemic baseline (relative to 6 January - 23 February 2020, shaded in gray), we isolate the percent change in health condition query proportions introduced during the pandemic where the differences between high- and low-income groups start to emerge. **e** We observe that low income ZIP codes experienced almost 200% less change in health condition queries compared to that of the high-income groups right after the US national emergency is declared (*n*_treated_ = 12,555, *n*_control_ = 3854). **f** When the same matching-based comparisons are performed across all SDoH factors during the first four weeks since the declaration of the pandemic in the US, ZIP code groups with lower incomes, higher proportions of Hispanic residents, higher population densities, and higher unemployment rates show significantly lower change proportions, while ZIP code groups with lower educational attainment show a significantly higher change in health condition query proportions (1603 ≤ *n* ≤ 12,575, Supplementary Table 6 for sample sizes per SDoH factor). In **e** and **f**, data are presented as mean values, and error bars indicate 95% confidence intervals. Both mean values and confidence intervals are obtained through bootstrapping with 500 iterations.

We divide our dataset according these SDoH factors and compare the magnitude of change in search behaviors between two ZIP code groups during the pandemic, where larger observed difference in the magnitude of change in search behaviors could indicate that one group’s response to the pandemic is more significant than the other in the level of interest in online information (e.g., health, unemployment) or in accessing online resources (e.g., online remote learning). For example, we split our ZIP codes into low and high-income groups (below and above *$*55,224 median household income) and compare the magnitude of change in health condition information queries (Fig. 1a). To disentangle the confounding effects of SES and race/ethnicity proportions on behaviors and health^63^, we compare changes in search behaviors on matched pairs of ZIP codes that are highly similar across these potentially confounding factors (Methods). We isolate the relative changes in search behaviors that occur concurrently with the pandemic using a difference-in-differences approach^64^, adjusting for yearly and weekly seasonality and for pre-existing, pre-pandemic disparities in query volume (Fig. 1b–d, Methods). Thus, we measure the disparate intensification or attenuation of search behaviors during the pandemic between the two ZIP code groups delineated by their distribution in a single SDoH factor (Fig. 1e). Finally, we apply the same process across all SDoH factors (Fig. 1f, Methods).

## Results

### Health information access

First, we examine the proportion of queries relating to a variety of health conditions (e.g., coronavirus and other health conditions including cancer or diabetes). Because the coronavirus, as the underlying cause of the pandemic, is at the forefront of everyone’s minds, the relative change in queries related to health conditions is almost 1000% higher than the pre-pandemic baseline. If all things were equal, we would see the same volume of response (i.e., the same relative change in query proportions) across all ZIP codes. However, given the higher rate of pre-existing health conditions, documented disparities in healthcare access, and higher COVID-19 case and mortality rates for low SES subpopulations^4,63^, we would expect to see that ZIP codes characterized by low SES would experience a greater intensification in their need for health information across a variety of health conditions and therefore increase their level of health information-seeking behaviors more than their counterpart ZIP code groups. Instead, we find that ZIP codes associated with lower incomes show over a 200 percentage point smaller increase (95% CI [−287, −152]) in health condition queries than their higher income counterparts (Fig. 1e). This means that a ZIP code that was yielding a thousand health condition queries per month before the pandemic makes about ten thousand such queries per month during the pandemic, but a similar ZIP code would only yield about eight thousand such queries per month if that ZIP code had lower median household income. We find that ZIP codes with higher proportions of Hispanic residents, higher population densities, and higher unemployment rates also responded to the pandemic with lower relative change in their health condition queries during the first four weeks (Fig. 1f). While ZIP codes with high (i.e., above population-average) proportions of Black residents (≥12%) do not seem to be affected as much as those with high proportions of Hispanic residents during the first four weeks, their response is lower during the months of August to November (Supplementary Fig. 14g). On the other hand, we find that ZIP codes with lower educational attainment (≤21.1% with bachelor’s degrees) make over 70 percentage points more (95% CI [31, 117]) health condition queries compared to ZIP codes with higher educational attainment (Fig. 1f).

Prior research has shown that SES and demographics correlated with online health information-seeking behaviors, highlighting the digital divide in health information access^65,66^. This divide has serious consequences. Through effective online health information-seeking behaviors, individuals can potentially make better healthcare choices and enjoy better health and well-being as a result, thereby reducing health disparities^8,54,65,67^. Unfortunately, our results suggest that disadvantages underlying certain socioeconomic contexts of ZIP codes (e.g., income, higher proportions of minority residents) independently are associated with attenuated participation in online health information-seeking behaviors relative to their counterparts. According to prior digital divide research^54,68,69^, a gap in health information-seeking behaviors may exacerbate health disparities down the line.

### Economic assistance access

During economic hardships and especially during the pandemic, the internet can be an efficient way for governments and institutions to deliver interventions and can lower barriers to accessing economic assistance or welfare services (e.g., https://www.usa.gov/food-help provides a comprehensive list of resources for food assistance). Unfortunately, the pandemic imposes multi-layered barriers to accessing crucial economic assistance because low SES subpopulations are more likely to suffer economically from the pandemic^70^ and deprioritize improving digital access as a consequence^54^. To understand changes in economic search behaviors during the pandemic, we examine behaviors for accessing unemployment and financial assistance on the web.

**Fig. 2:**
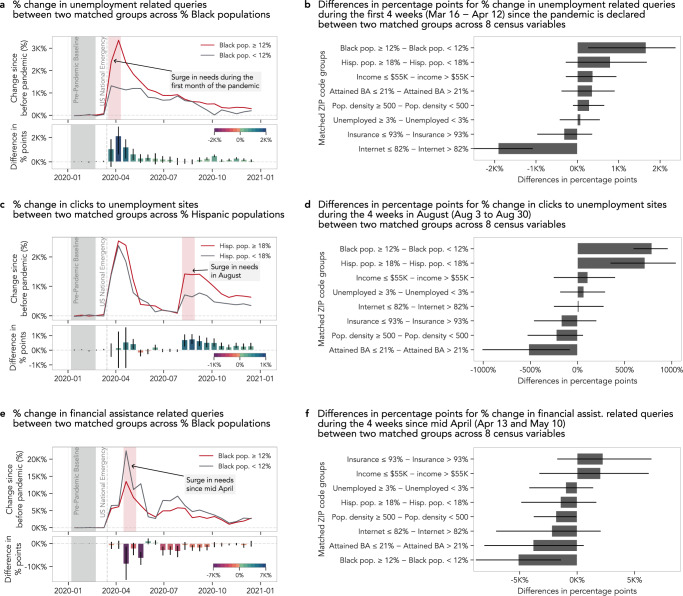
Disparities in online economic assistance access. **a** The surge in unemployment-related search queries peaks during the first month since the declaration of the pandemic and tapers off over the year 2020. During this first month, ZIP codes with higher proportions of Black residents (≥12%) have expressed up to 3,358% more unemployment-related queries while ZIP codes with lower proportions of Black residents (<12%) have expressed 1320% more. **b** Across the seven census variables, ZIP codes with higher proportions of Black or Hispanic residents and lower income populations experienced greater changes in unemployment-related queries during this first month. **c** When we examine search queries that led to clicks in state unemployment sites, we see a second surge in August, with ZIP codes with higher proportions of Hispanic residents (≥18%) experiencing more than double the change in clicks in state unemployment sites compared to ZIP codes with lower proportions of Hispanic residents (<18%). **d** We observe that ZIP codes with higher proportions of Black and Hispanic residents experience a greater change in clicks in unemployment sites during the month of August, but ZIP codes with low educational attainment express less change in clicks in unemployment sites. **e** Search queries related to financial stimulus were at their peak in late April, right after the time that the first stimulus checks were deposited on April 11. **f** However, throughout the year and especially during the four weeks since mid-April, ZIP codes with higher proportions of Black residents experienced a smaller change in financial stimulus-related queries than ZIP codes with lower proportions of Black residents. In all bar charts in **a**–**f**, data are presented as mean values, and error bars indicate 95% confidence intervals (1603 ≤ *n* ≤ 12,575, Supplementary Table 6 for sample sizes per SDoH factor). Both mean values and confidence intervals are obtained through bootstrapping with 500 iterations.

When we examine unemployment-related search interactions, we find that relative changes in unemployment-related search queries (e.g., “eligible for unemployment benefits”, “jobless claims”) closely follow those of reported unemployment claims by the Bureau of Labor Statistics (Supplementary Fig. 12a). However, the intensification of unemployment search queries in ZIP codes with higher proportions of Black residents is almost three times the increase corresponding to ZIP codes with lower proportions of Black residents (Fig. 2a), with a 3026% increase in query proportions for ZIP codes with higher proportions of Black residents compared to an over 1365% increase for their counterparts, resulting in a 1661 percentage point difference (95% CI [260, 2374]) (Fig. 2b).

Potential interest in digital unemployment resources is not captured in reported claims that measure unemployment claims that are actually submitted, but it can be readily observed in web search logs. For example, we find another surge in search queries that resulted in an over 1000% increase in the proportion of clicks on state-specific unemployment websites past July 2020 (Supplementary Fig. 12b), at which point the expanded federal supplement to unemployment insurance benefits expired (Fig. 2c). During the month of August, ZIP codes with higher proportions of Black and Hispanic residents present 789 (95% CI [595, 957]) and 716 (95% CI [351, 1043]) percentage points more in their change in clicks to unemployment sites, indicating that ZIP codes with higher proportions of Black and Hispanic residents may have required additional long-term unemployment benefits. Conversely, ZIP codes with lower educational attainment levels experienced 517 percentage points less (95% CI [−1009, −81]) in the change in state unemployment site visits (Fig. 2d). Such discrepancy between interests in unemployment benefits expressed online and officially submitted claims and the relatively attenuated access to such resources may suggest potential barriers in the successful submission of benefit applications (e.g., confusion, eligibility^28,29^). Coupled with a low recipiency rate of unemployment benefits^71^ and the association between unemployment accessibility and suicide risks^72^, the mismatch between demands and claims is concerning.

April of 2020 was a prime occasion for financial assistance-related queries (e.g., “loan forgiveness”, “stimulus check deposit”) because the first stimulus checks were deposited on 11 April 2020 (Fig. 2e). We find that financial assistance-related queries increased by over 15,000% in mid-April on average, but ZIP codes with higher proportions of Black residents experience 5,119 percentage points less change (95% CI [−8809, −1407]) in financial assistance-related queries between 13 April and 10 May 2020 (Fig. 2f). That means that if a ZIP code yielded 100 financial assistance-related queries per month in mid-April of 2019, that ZIP code yields 16,700 such queries per month in mid-April during the pandemic, but only 11,600 queries for an otherwise similar ZIP code with a higher proportion of Black residents. Since we successfully controlled for other potential confounding factors such as income and educational attainment in our comparison, as shown in Supplementary Table 8, our result points to higher proportions of minority residents within ZIP codes, not necessarily the racial composition of the ZIP codes per se and certainly not the race/ethicity itself, as a plausible source for such disparity. Our finding highlights the need to further investigate potential barriers or disadvantages unobserved in our data that disproportionately prevent ZIP codes with higher proportions of Black residents from responding to pandemic-induced stimulus demands on the web.

### Shift to digital learning and food delivery resources

The COVID-19 pandemic brought a rapid and massive digital transformation to lives as mandated lockdowns forced people to transform and reimagine traditional interpersonal connections (e.g., going to school, getting food, or meeting friends) into virtual digital ones. Unfortunately, digital inequalities worsen social and material deprivations and perpetuate existing disadvantages into a digital vicious cycle^10,73^. To observe changes in education search behaviors during the pandemic that may be useful to understand this vicious cycle, we investigate two types of digitally mediated activities that would be presumed to be particularly sensitive to pandemic-induced limitations on in-person access: online remote learning and online food delivery services.

**Fig. 3:**
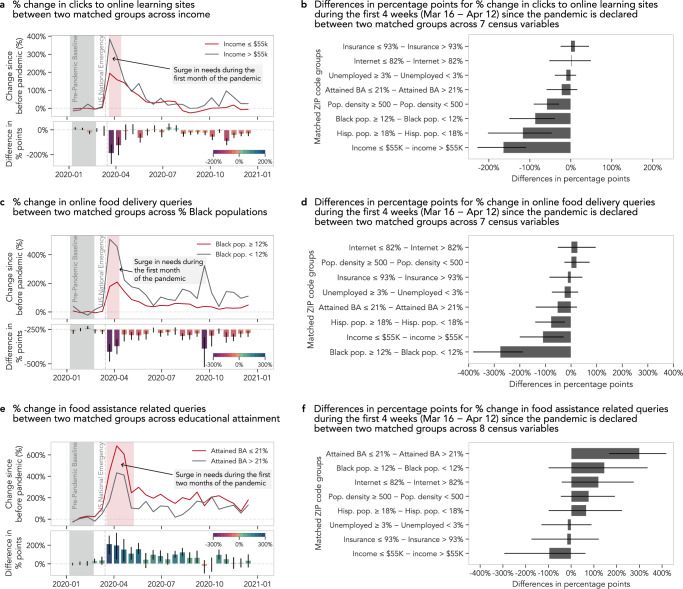
Disparities in shifting to digital resources. **a** Online learning sites played a significant role in filling in the gaps introduced by school closures at the beginning of the pandemic with an over 200% increase in engagement. **b** However, ZIP codes with lower income and higher proportions of Black or Hispanic residents experienced smaller changes in queries that resulted in clicks to online learning sites during the first month. **c** With mandated lockdowns, populations have transitioned to food delivery services during the pandemic, but the rate of change in online food delivery queries is more than twice for ZIP codes with lower proportions of Black residents. **d** We see that ZIP codes with higher proportions of Black or Hispanic residents or lower income experienced a smaller change in online food delivery services during the first month of the pandemic. **e** Food assistance-related queries were also in high demand with an over 400% increase at the beginning of the pandemic. **f** ZIP codes with lower educational attainment experienced a greater change in food assistance-related queries than ZIP codes with higher educational attainment. In all bar charts in **a**–**f**, data are presented as mean values, and error bars indicate 95% confidence intervals (1603 ≤ *n* ≤ 12,575, Supplementary Table 6 for sample sizes per SDoH factor). Both mean values and confidence intervals are obtained through bootstrapping with 500 iterations.

Statewide mandates in the US required many schools to close in-person learning as early as 16 March 2020^74^, and school districts scrambled to implement remote learning alternatives. Many parents, students, and teachers turned to free online resources such as Khan Academy to fill the gaps temporarily or permanently^75^. There were also reported disparities in access to technologies or live virtual learning as well as absenteeism that stymied low-income students^76^. When we examined search queries that result in visits to free online learning resources (e.g., coursera.org, khanacademy.org), during the first four weeks of the pandemic, there was an overall increase in the proportion of queries that led to online learning sites compared to before (seen as a positive percent change in Supplementary Fig. 21). During this time, we found that ZIP codes with lower income and higher proportions of Hispanic residents exhibited only half to two-thirds of the increase (percentage point difference 95% CI [−227, −109] and [−202, −46], respectively) in those queries relative to their counterpart groups (Fig. 3a). If a ZIP code yielded 100 search-led clicks to online learning sites per month before the pandemic, that same ZIP code would yield 500 such clicks per month during the pandemic, but only 300 such clicks would be observed for a similar ZIP code with lower income or a higher proportion of Hispanic residents, even after controlling for internet access (Fig. 3b). ZIP codes with higher proportions of Black residents and higher population densities exhibit a similar trend. Even though these free online learning resources are designed to be accessible and flexible, helping students to go at their own pace, we find that ZIP codes with low-income or high proportions of Black or Hispanic residents did not leverage them at the same level as their counterpart ZIP code groups during the pandemic.

On the other hand, during the fall academic period of 2020, the proportion of queries that led to online learning sites decreased compared to before (seen as a negative percent change in Supplementary Fig. 21). During this time, we found that ZIP codes with lower income and higher unemployment rates exhibited a smaller attenuation (i.e., their change remained closer to the baseline, Supplementary Fig. 22e, h), but ZIP codes with a higher proportion of Black residents exhibited a larger attenuation (Supplementary Fig. 22g).

In addition, school districts in low SES neighborhoods were more likely to be closed during the pandemic and less equipped to provide remote learning or at-home assignments, greatly reducing opportunities for both in-person and online learning for students with negative educational outcomes^77,78^. Our findings suggest that there exists unintended consequences of the public health policies that perpetuate a myriad of disadvantages, as education is such a crucial factor in digital literacy^12,79^, income^80^, and health^54,81^.

COVID-19 fundamentally changed people’s purchasing and spending behaviors, as many of the restaurants, stores, and non-essential businesses were closed to in-person shopping^82^. Spending on food delivery and groceries also increased significantly during the pandemic, with more people eating at home with a higher utilization of online e-commerce platforms for accessing food and groceries^82,83^. When we examine search queries for online food delivery (e.g., “grocery delivery”, “deliver food”), we find that online food delivery queries increased by over 500% for ZIP codes with lower proportions of Black residents while those with higher proportions of Black residents only increased by over 170% (percentage point difference 95% CI [−382, −188], Fig. 3c, d). We found similar lessened engagement in online food delivery searches for ZIP codes with lower income and higher proportions of Hispanic residents (95% CI [−200, −29] and [−140, −24], respectively, Fig. 3d). These findings could be explained by the fact that low-income subpopulations receive and seek more food assistance and tend to eat food away from home less frequently^84^ and that such online food delivery services may not be accessible because they incur higher costs for consumers, given the markup and delivery surcharges.

ZIP codes with lower educational attainment also experienced a 301 percentage point higher increase (95% CI [167, 419]) in queries for seeking food assistance (e.g., “Supplemental Nutrition Assistance Program", “help with food stamps”, “free and reduced lunch”, Fig. 2e, f) relative to their highly educated counterparts. Unfortunately, those that relied on these traditional food assistance programs were left with severely limited choices during the pandemic because these programs do not extend to online purchase or delivery services^85^. Our findings highlight a potential gap between the increased food assistance need, as illustrated by the increase in the online information-seeking behavior about food assistance, and the ability to actually procure food goods through online food purchase and delivery services.

## Discussion

We conducted a longitudinal study during the pandemic to observe the second-level digital divide at population scales. Specifically, we leveraged the centrality of web search engines for online information access to quantify how offline exclusion relates to the intensification and attenuation of existing digital exclusion during the pandemic. Our use of search data provided a near real-time and unique lens into naturalistic digital behaviors^31^. Our analysis revealed potentially unmet needs that are unobserved by other data sources. For example, we observed a surge in unemployment site visits in August 2020 that are not captured by the unemployment claims data. We also observed differential uses of pandemic-relevant online resources that span health information, learning, and food delivery. Most importantly, we demonstrated a disproportionate change in a community’s use of these digital resources across several socioeconomic and environmental offline factors. These differences are significant when put into the context of the bidirectional nature of digital and offline exclusion where the lack of the ability to capitalize on digital resources could lead to negative downstream offline outcomes^53,56,57^.

Our study is structured around the SDoH, a framework commonly used and well-known in public health and disparities research, not only to cover a broad spectrum of factors but also to identify opportunities that promote future research around specific determinants. Under the Economic Stability determinant, we found that the lack of economic stability (median household income, % unemployment) are associated with a smaller increase in engagement in health information-seeking behaviors or online learning consumption at the onset of the pandemic, compared to their higher-income counterparts. Under the Social and Community Context determinant, we found that ZIP codes with higher proportions of minority residents (% Black residents, % Hispanic residents) exhibited smaller increases in health information-seeking, online learning, or online food delivery behaviors, indicating that these groups fell behind in the digital shift catalyzed by the pandemic^10^. Unemployment-related queries were increased the most by ZIP codes with higher proportions of Black residents at the onset of the pandemic. Unemployment-related site visits were increased the most by ZIP codes with higher proportions of Hispanic residents beyond August, indicating a second wave of potentially unmet demand for unemployment assistance. Under the Education Access and Quality determinant, we found that lower educational attainment (% with BA or higher) are associated with a larger increase health information-seeking and food assistance-seeking behaviors. Under the Neighborhood and Built Environment determinant, we found that higher population density is associated with a smaller increase in health information-seeking and online learning behaviors. Although internet access was not a variable we examined through matching, per our focus on the second-level divide, we found that the lack of internet access does associate with lower unemployment queries. Because we controlled for all other SDoH factors when comparing groups delineated by a single SDoH factor, our findings have implications for designing determinant-specific interventions and also for examining their potential long-term impacts. Although there are factors we did not find to be significant (e.g., % with healthcare), we caution against interpretations of such factors or interventions not being useful or necessary.

Our analysis along the SDoH factors probes into plausible sources of disproportionate digital behaviors only at ZIP code levels, and understanding the disadvantages underlying these factors and mechanisms for such disparities that permeate through the life course of an individual must be further investigated. In recent years, the SDoH has been referenced in relation to digital divide; digital literacy and internet access are referred to as super determinants of health as they relate to all social determinants of health^86^. Recent digital divide literature also raises an alarm for third digital divide (i.e., the differential offline outcomes that people obtain from their use of digital technologies) and highlights the important interplay between different levels of divide as well as the role of digital capital in bridging online and offline realms^53,56^. Therefore, our findings frame important research questions on the downstream real-world implications of differential information search behaviors. For example, high priorities must be assigned in understanding the long-term offline impacts of low-income communities not leveraging as much online learning resources or communities with higher proportions of Hispanic residents having intensified unmet demands for online unemployment assistance compared to their counterpart groups. Although the SDoH factors and outcomes reviewed in our analysis are generally not modifiable (e.g., race) or difficult to modify (e.g., income), our findings nevertheless highlight specific at-risk populations for whom to target shielding or interventions^87^.

Prior studies have shown that access to digital resources and information and the incorporation of such digital technologies in everyday lives from childhood are crucial for upwards mobility^54^. Although SES is an important factor in shaping disparities in digital access, prior research has shown that SES also impacts levels of web expertize and the utilization of digital resources for information-seeking activities^55^. Low SES populations suffer from the lack of training and educational support key to building the necessary skills to make efficient use of digital access and tools^12^, highlighting that simply making the internet more accessible may not level the playing field^88^. In the context of the current COVID-19 pandemic, where digital access and resources became more critical due to prolonged at-home isolation and restrictions on in-person activities, communities characterized by low SES may experience the compounding effects of multiple potential disadvantages that may manifest as disparate reactions to the pandemic in digital engagement.

We note the inherent limitations of studying digital engagement using digitally obtained data: This and other studies with online data can inadvertently exclude those who leave no or very little digital footprint^54^. Our information sources provide signals about levels of activity, but we cannot study the details of changes in types of access if there is no engagement. Our analysis is also limited to the footprint of Bing as one of several search engines used for online information access, and Bing’s user population may not be fully representative of the United States population. We use both English regular expressions as well as language-independent click-based measures, but did not include regular expressions in other languages. Our study carefully controls for internet access, as measured by the census, such that any observed effects cannot be explained by differences in internet access across ZIP code groups. Our observed changes can only be attributed to ZIP code levels and not individuals because individual-level SDoH factors are not available and to preserve anonymity. Our work provides a holistic characterization of digital engagement using broad categories spanning health, economics, education, and food, and we cannot make claims about specific subcomponents (e.g., individual keywords). Our longitudinal comparison between before and during the pandemic cannot be used to isolate the changes in search behavior to be solely attributable to the pandemic to make any causal claims, despite our adjustments for temporal variations.

Our current data cannot be directly used to discern whether different access behaviors are due to the lack of web expertize (i.e., digital literacy or search facility), the lack of awareness of the value of information (i.e., attitude towards information), or the lack of intangible resources like time and energy. However, concepts like digital literacy, which is an important factor in the embodiment of digital capital, can be quantified by careful examination of an individual’s search behavior. As prior research has shown, search interactions vary, based on the user’s familiarity with search engines or their domain expertize^89,90^. Quantifying digital literacy combined with a longitudinal observation of socioeconomic and environmental factors could provide empirical evidence for how digital literacy operates in the attainment of offline economic, cultural, and social capitals^53^, and our large-scale, search-based methodology opens the doors of opportunities for monitoring such phenomena. In addition, we see value in follow-up, small-scale focused studies aimed at contextualizing individuals’ experiences of the crisis and measuring the effects of community-specific interventions^10^. These community-specific interventions could include raising the level of digital literacy (e.g., education around web expertise or digital know-how) or improving the quality of digital access (e.g., high-speed, uninterrupted internet access or high-end equipment). Quality of access, especially through different device types or device specifications, has been highlighted as another important factor in recent digital divide research^13^. Therefore, more work is needed to understand the differential uses on desktop or mobile devices. These may also include non-digital methods because traditional methods (e.g., text messaging, handouts) have been shown to work better for low SES populations^76^. Future research aimed at understanding digital disparities, therefore, must acknowledge the correlations between different SES, race/ethnicity, and social determinants of health^91^ and leverage methods that embraces their interrelatedness^92^.

This study presents a web-based approach to understanding digital disparities. It demonstrates that web search logs can be harnessed to characterize and deliver key insights about the disproportional utilization of digital resources to meet everyday needs during global crises. Our observational study design is able to scale to a large population (billions of queries by millions of people) to quantify the disparities in digital engagement. Building on prior disparities research that advocated for a comprehensive look at SES factors including race/ethnicity^63,91^, our study emphasizes the inclusion of a broad set of factors and outcomes representative of the SDoH. Through the lens of SDoH factors, our findings highlight disadvantaged communities that may be struggling to overcome burdens induced by the pandemic and have disproportionately intensified or reduced their access to critical online resources. Therefore, future public health interventions should target both potential barriers to access that pull communities away from necessary digital resources as well as provide support to ensure that the intensified need for digital resources are adequately met.

## Methods

### Data set and study population

Our source dataset consists of a random sample of 57 billion de-identified search interactions in the United States from the years 2019 and 2020 from Microsoft’s Bing search engine. Each search interaction includes the search query string, URLs of all subsequent clicks from the search result page, timestamp, and ZIP code. We excluded search interactions from ZIP codes with less than 100 queries per month so as to preserve anonymity. Our search dataset intentionally includes both desktop and mobile Bing search interactions in order to capture both search query sources. Although the quality of access, especially through different device types or device specifications, has been highlighted as another important factor in recent digital divide research^13^, analysis on the differential search behaviors across device types is outside the study’s scope. All data were de-identified, aggregated to ZIP code levels or higher, and stored in a way to preserve the privacy of the users and in accordance to Bing’s Privacy Policy.

While many Americans use other search engines such as Google, Bing’s query-based market share is estimated to be ~26.7% according to Comscore data^93^. We focused on query-based metrics for estimating search market share because it captures end-users’ interaction with the search engine, including queries that may not have resulted in site visits. Click share, on the other hand, captures only search-driven traffic to a subset of websites that are instrumented with custom code. To understand the validity of relying solely on Bing search data, we compared Bing and Google queries for matched categories longitudinally and found that the search trends are highly correlated (Pearson *r* = 0.86 to *r* = 0.98, Supplementary Fig. 2). Our search ZIP code data is provided by a proprietary location inference engine, with added accuracy improvements to standard reverse IP lookup databases from contextual and historical information, but such estimation is still an approximation. Our study also assumes that the demographics of the search users in a ZIP code reflect the demographics of the population within a ZIP code. However, search users generally trend towards more white, richer, and older population. It is difficult to accurately characterize the population base without third-party services such as Comscore data^93^, which may have its own limitations and biases. Our analysis of a proportion of user demographics using such data confirms that Bing data tracks the US population reasonably well.

The study (protocol ID 632) was reviewed by the Microsoft Research Institutional Review Board (OHRP IORG #0008066, IRB #IRB00009672) prior to the research activities. Microsoft Research is an industry-based research institution with a United States Department of Health, Human Services (HHS) federally registered IRB. In addition to following federal ethical research guidelines, Microsoft Research IRB takes an anthropological stance in looking at the impacts of research and looks beyond the risks to human subjects, according to IRB regulations, but also risks to human society^94^. The authors and the Microsoft Research IRB recognize the sensitive nature of the use of data collected from Microsoft users for research purposes. Our study followed the privacy and security regulations governed by Microsoft’s privacy statement as well as the federal ethical guidelines set forth by the HHS. All search data have been de-identified and aggregated prior to receipt by our study team such that no identifiable information was processed or analyzed. Via a standard ethical review process prior to the study, Microsoft Research IRB formally approved our study as “Not Human Subjects Research” to indicate that the activities do constitute research, but where the definitions of “human subject” and “identifiable private information” do not apply (as defined by 45*§*46.102(e)). Microsoft Research IRB certifies that our Human Subjects Review process follows the applicable regulations set forth by the Department of Health and Human Services: Title 45, Part 46 of the Code of Federal Regulations (45 CFR 46) (the Common Rule), and our Ethics Program promotes the principles of the Belmont Report in our research institution. In addition to the ethics review, our study obtained approvals from Microsoft’s privacy, security, and legal review officers prior to obtaining and analyzing the data.

#### ZIP code level data

One of our goals is to characterize the role of socioeconomic and environmental factors on digital engagement outcomes. Unfortunately, data that combines individual-level search interactions with each individual’s socioeconomic and environmental characteristics at the US national scale does not exist, is difficult to capture, and invites privacy concerns. Instead, we use ZIP codes as our geographic unit of analysis. ZIP code level analysis can be limited because it cannot describe each individual living in those ZIP codes. However, ZIP code level analysis can scale to nontrivial population sizes and has been repeatedly recognized and leveraged in population-scale and local/neighborhood-level research^20,95–100^. ZIP code level analysis also enables accounting for well-known issues associated with residential segregation and socioeconomic disparities^12,101^. We leveraged the available ZIP code level American Community Survey estimates using the Census Reporter API^102^ in order to characterize the ZIP codes in our dataset.

#### Census variables and search categories

We chose a set of census variables to delineate ZIP code groups as well as search categories to examine digital behaviors. Supplementary Fig. 1 illustrates our full choice of census variables and search categories.

The SDoH has been widely used as a holistic framework to describe a broad range of socioeconomic and environmental factors that determine one’s health, well-being, and quality of life. In recent years, the SDoH has also been referenced in relation to digital divide; digital literacy and internet access are referred to as super determinants of health as they relate to all social determinants of health^86^. Just as Helsper^57^ theorized the corresponding digital and offline fields, looking at variables from both offline and digital aspects of the social determinants of health are critical in understanding digital disparities. Because of the multidimensional nature of socioeconomic status and its association with health and well-being outcomes, it is important to include relevant socioeconomic factors^91^. Therefore, our choice of census variables and search categories are largely influenced by the SDoH framework defined by the US Department of Health^62^.

We considered multiple socioeconomic factors including race, income, unemployment, insurance coverage, internet access, educational attainment level, population density, age, gender, Gini index, homeownership status, citizenship status, public transportation access, food stamp, and public assistance. We did not include some of the factors when they were highly similar to already included factors (e.g., % below poverty level is correlated to median household income, Pearson *r* = − 0.624). In the end, we included eight census variables that represent all five categories of SDoH to cover a broad range of socioeconomic and environmental factors.

Under Healthcare Access and Quality, we included the percentage of the population with health insurance coverage (Table B27001). Under Education Access and Quality, we included the percentage of the population that attained a Bachelor’s degree or higher (Table B15002). Under Social and Community Context, we included the percentage of the population of Hispanic origin (Table B03003) and the percentage of the population with Black or African American alone (Table B02001). Under Economic Stability, we included the median household income (Table B19013) and the percentage of the civilian labor force that is unemployed (Table B23025). Under Neighborhood and Built Environment, we included the percentage of the population with a broadband or dial-up internet subscription (Table B28003) and the population density. We computed per ZIP code population density by joining area measurements from ZIP Code Tabulation Areas Gazetteer Files^103^ and total population (Table B01003). We joined the search interaction data with the above SDoH factors on ZIP codes and excluded ZIP codes that did not have either search interactions or census data. The resulting 55 billion search interactions covered web search traffic from 25,150 ZIP codes in the US, and these ZIP codes represent 97.2% of the total US population. Supplementary Table 1 provides per-ZIP code summary statistics of our dataset.

Our choice of search categories was largely informed by our prior work^9^. We chose three determinants—Healthcare Access and Quality, Education Access and Quality, and Economic Stability—from which to draw our search categories. We excluded two determinants that were generally more difficult to capture with simple query string matches because they tend to be more contextual (e.g., location, social) than can be expressed as query strings for information-seeking. Under the three SDoH factors, we chose seven search categories that not only appeared more frequently than others in our dataset but also were relevant topics during the pandemic. Supplementary Table 4 enumerates the categories we examined with example query strings, URLs, and regular expressions.

Examining individual search keywords or subcategories has been pursued by others within and outside the scope of the pandemic. In our study, the use of broad categories spanning health, economics, education, and food is intended to capture a holistic view of the pandemic across many different needs^61^. Accordingly, we do not make any claims about subcomponents within a category because studying these subcomponents is out of scope of this work.

Certainly, there exist search keywords that are more popularized by the current pandemic, such as “coronavirus” or “covid”, that also belong in the health information category. However, these keywords are not unique to the current pandemic and have existed before. As infrequent searches for “coronavirus” might seem in 2019, in our data, the query frequency of “coronavirus” in 2019 was similar to that of “mers” and certainly not zero (Supplementary Fig. 3). In fact, many categories of interests exhibited changes during the pandemic^9,19^, not just some that are highly relevant to the pandemic. For example, Suh et al.^9^ has demonstrated that many of the ordinary search topics, such as “toilet paper”, “online games with friends”, or “wedding” were significantly changed during the pandemic.

### Disproportional change in digital engagement during the pandemic

Our goal is to quantify the disproportional change in digital engagement during the pandemic experienced by different subpopulations. Our study conducts several data processing steps and analysis methods to arrive at our findings: (1) we quantify digital engagement by computing relative query proportions for various search categories, (2) we quantify intensification or attenuation of digital engagement by computing changes in digital engagement between before and during the pandemic, and (3) we compare the changes in digital engagement across ZIP code groups.

#### Digital engagement trends

We leverage interactions with search engines to obtain signals about digital engagements where everyday needs are expressed or fulfilled through a digital medium, in our case Bing^9^. In our study, we characterize digital engagement through modeling users’ search interests as expressions of underlying human needs^9^, building upon prior work that uses search interactions to model interests that are either expressed explicitly through search queries or implicitly through clicks on results displayed on the search engine result page^16–18^. To gain a nuanced understanding of these search interactions, we categorize each search interaction into topics ranging from health access, economic stability, and education access. We match each search interaction to a corresponding category through simple detectors based on regular expressions and basic propositional logic (Supplementary Table 4). Each category could have multiple regular expressions applied to either the query string, the clicked URL, or both. Then, we count matching search interactions for a given category. Our query string detectors operate only on English-language keywords such that any cross-cultural or cross-language analysis is out of scope of this work, but some of our detectors include looking at the click results regardless of the query.

To capture the level of search interest in these categories in relation to all other categories of interest, we compute the proportion of total search queries that belongs to a specific category. For example, we compute the proportion of total search queries that contain health condition keywords such as cancer, diabetes, or coronavirus to quantify the level of interest in engaging in health information-seeking behaviors in relation to all other digital engagement behaviors. In another case, we examine search queries that result in subsequent clicks to state unemployment benefit sites to quantify the level of interest in unemployment benefits.

In addition, the focus on the level of interest through query proportions rather than query frequencies is helpful in our analysis. First, it helps with accounting for the baseline differences in search access between two populations. Second, this focus on relative measures of search query frequency helps adjust for changes in query volume over time, which is a common practice in Information Retrieval and web search log analysis^104,105^. Supplementary Fig. 4–11 illustrate the temporal variations in relative query frequencies (left) and in relative query proportions (right) in each query category for each of the two matched groups across all SDoH factors. Adjusting for the baseline differences in search access allows us to remove the existing access differences between the two groups, and the temporal trends of the query proportions between the two groups become much closer.

#### Longitudinal before and during pandemic change in digital engagement

To capture longitudinal changes in search behaviors that are most likely attributable to the pandemic, we use a difference-in-differences (DiD) method^64^ to apply several corrections. DiD is often used in econometrics and public health research as a quanti-experimental research method to study causal relationships where a randomized control trial (RCT) is infeasible^106^. Using DiD design with the pandemic as the treatment cannot lead to any causal claims because there is no control group or a counterfactual (i.e., everyone is exposed to the pandemic). In our study, we leverage DiD method to quantify the intensification or attenuation in search behaviors by removing seasonal variations and normalizing on pre-pandemic baselines.

After we categorize each search interaction with our categories of interest, we count and aggregate them per time window (i.e., 2-week or 4-week intervals in our analysis) and per ZIP code (Fig. 1a). We compute the proportion of the total query volume represented by each category for these time windows to quantify the level of search interests in that category while removing undesired variations in the query volume over time (Fig. 1b). We denote the digital engagement at time *t* in category *c* as the fraction of the total number of queries at time *t*: *E*(*t*, *c*) = *N*(*t*, *c*)/*N*(*t*). From this, we control for yearly seasonal variations by subtracting the digital engagements of 2019 from that of 2020: *E*(*t*^2020^, *c*) − *E*(*t*^2019^, *c*). People tend to behave differently on weekends, and we observed a 7-day periodicity in our data, sometimes known as the “weekend effect”^107^. Therefore, when comparing two years, it is important to account for the weekend effect. In order to highlight the actual differences that are not explained by weekend mismatches across years, we aligned the day of the week between both years (i.e., Monday, 6 January 2020 is aligned to Monday, 7 January 2019). In addition, we ensured that our comparison analysis included all seven days of the week (i.e., look at means across one or multiples of a full week) (Fig. 1c).

Finally, to compute the change in digital engagement during the pandemic since the time at which the US national emergency was declared on 16 March 2020, we subtract the query proportions between 6 January 2020 and 23 February 2020, a period we defined as the “pre-pandemic baseline” (Fig. 1d). Even though the national emergency was declared three weeks later, we use 23 February 2020 as the cutoff because individual states declared a state of emergency at different times between February 29 and March 15 of 2020 and to avoid partial weeks in our analysis. Our estimate of the *relative change in digital engagement* in category *c* between before and during the pandemic is defined as:

1 $$C({t}_{{{{{{{{\rm{before}}}}}}}}};{t}_{{{{{{{{\rm{during}}}}}}}}},c)=\left[E\left({t}_{{{{{{{{\rm{during}}}}}}}}}^{2020},c\right)-E\left({t}_{{{{{{{{\rm{during}}}}}}}}}^{2019},c\right)\right]\\ -\left[E\left({t}_{{{{{{{{\rm{before}}}}}}}}}^{2020},c\right)-E\left({t}_{{{{{{{{\rm{before}}}}}}}}}^{2019},c\right)\right]$$

Or the relative *percentage change* in digital engagement *C*_perc_ is expressed as:

2 $$	{C}_{{{{{{{{\rm{perc}}}}}}}}}({t}_{{{{{{{{\rm{before}}}}}}}}};{t}_{{{{{{{{\rm{during}}}}}}}}},c)\\ 	=\frac{\left[E\left({t}_{{{{{{{{\rm{during}}}}}}}}}^{2020},c\right)-E\left({t}_{{{{{{{{\rm{during}}}}}}}}}^{2019},c\right)\right]-\left[E\left({t}_{{{{{{{{\rm{before}}}}}}}}}^{2020},c\right)-E\left({t}_{{{{{{{{\rm{before}}}}}}}}}^{2019},c\right)\right]}{\left[E\left({t}_{{{{{{{{\rm{before}}}}}}}}}^{2020},c\right)-E\left({t}_{{{{{{{{\rm{before}}}}}}}}}^{2019},c\right)\right]}\times 100$$

We acknowledge that there may exist a ZIP code with zero or very little search interactions for a given category, especially before the pandemic and in 2019. For example, “stimulus check” may only be relevant during the pandemic for certain ZIP codes. We cannot exclude these ZIP codes because we want a good representation and distribution of ZIP codes in our analysis. If a ZIP code makes only a handful of search queries on various health conditions, for example, but the number of queries increases dramatically due to concerns surrounding comorbidities and health complications, that is precisely the signal we hope to capture and observe across ZIP code groups. We mitigate this potential challenge of zero or near-zero baseline issues in several ways. (1) Our regular expressions are inclusive of potential variations in expressing the categories, including expressions that are likely to occur before the pandemic and in 2019. (2) We aggregate search interactions in two or 4-week windows, which consequently reduces the likelihood of having no or very little search interaction before the pandemic. (3) We also aggregate across thousands of ZIP codes that belong to a specific group (e.g., a group of ZIP codes with median household income greater than *$*55,224), where the likelihood of having no or very little search interaction before the pandemic for each group is 0%. (4) Instead of computing per-ZIP code DiD, we compute per-group DiD. In other words, we perform a within-group summation before taking the difference, which allows us to characterize the change in digital engagement for a typical ZIP code in the group.

#### Comparisons across ZIP code groups

Finally, we aggregate these changes in digital engagements across two comparison ZIP code groups for each SDoH factor, for example, to compare the average change in digital engagement of low-income ZIP codes with the average change of the high-income ZIP codes (Fig. 1d). Thus, we operationalize the disproportional change in digital engagement during the pandemic by quantifying the differences in the changes in search behaviors for a single search category between two ZIP code groups delineated by a single SDoH factor (Fig. 1). In our analysis, we report the change in digital engagement as the percentages of the pre-pandemic baseline, *C*_perc_, where 0% denotes no change. We report the disparities in the changes in digital engagement between two comparison ZIP code groups as the percentage point difference where 0 denotes no difference (Fig. 1e, f). We formalize disparities in the changes in digital engagement in category *c* during the pandemic between high-risk ZIP code group *g*_high_ and low-risk ZIP code group *g*_low_ as:

3 $${D}_{{{{{{{{\rm{perc}}}}}}}}}({t}_{{{{{{{{\rm{before}}}}}}}}};{t}_{{{{{{{{\rm{during}}}}}}}}},{g}_{{{{{{{{\rm{low}}}}}}}}};{g}_{{{{{{{{\rm{high}}}}}}}}},c)={C}_{{{{{{{{\rm{perc}}}}}}}}}^{{g}_{{{{{{{{\rm{high}}}}}}}}}}({t}_{{{{{{{{\rm{before}}}}}}}}};{t}_{{{{{{{{\rm{during}}}}}}}}},c)\\ -\,{C}_{{{{{{{{\rm{perc}}}}}}}}}^{{g}_{{{{{{{{\rm{low}}}}}}}}}}({t}_{{{{{{{{\rm{before}}}}}}}}};{t}_{{{{{{{{\rm{during}}}}}}}}},c)$$

To obtain non-parametric 95% confidence intervals, we conducted bootstrapping with replacement at 500 iterations during this aggregation step. These confidence intervals are computed when estimating the effect size (i.e., the difference between matched groups) and are visualized with figures demonstrating the difference between groups. All errors bars in figures indicate this 95% bootstrapped confidence interval (*N* = 500). Supplementary Figs. 13–26 illustrate percent changes in each query category for each of two matched groups and their differences in percentage points across all SDoH factors.

### Matched comparison groups

Our goal is to quantitatively estimate the independent association between one socioeconomic factor and the changes in digital engagement while controlling for other factors during a global crisis such as the COVID-19 pandemic. Specifically, we are interested in eight SDoH factors: (1) median household incomes, (2) % unemployed, (3) % with health insurance, (4) % with Bachelor’s degree or higher degrees, (5) population density, (6) % Black residents, (7) % Hispanic residents, and (8) % with internet access.

One way to do this is to conduct a simple univariate comparison between the two groups. However, one would quickly realize that the high-income group has a fewer minority race than the low-income group, making the comparison biased. Many of the socioeconomic and racial variables are known to be correlated^63,91,108^. This means that univariate analysis of outcomes along one SDoH factor would likely be confounded by multiple other variables. In fact, within our dataset, we observed high correlation among many SDoH factors examined (Supplementary Table 3). For example, the median household income of the ZIP codes in our dataset is negatively correlated with the percentage of Black residents (Pearson *r* = − 0.23) and is positively correlated with internet access (Pearson *r* = 0.66). Comparing high and low-income groups without considering other factors would result in two groups of uneven distributions of race and internet access, among many other factors. Therefore, it is important to consider these factors jointly and adequately control for SES factors when analyzing outcome disparities^63,91^. To create a comparable and balanced set of groups with similar covariate distributions, we leverage matching-based methods.

Matching-based methods are commonly used to replicate randomized experiments as closely as possible in situations when randomized experiments are not possible from observational data^109,110^. This is achieved by obtaining balanced distribution of covariates in the treated and control groups^109,111^. Even though matching-based methods are commonly used for causal inferences, the same matching-based method can also be used to answer noncausal questions^109^ (e.g., racial disparities^112^). Our study, therefore, performs a longitudinal before-after observational study with matched groups to answer noncausal questions of the form: How did the changes in search behaviors during the pandemic differ across matched groups delineated by a single socioeconomic and environmental factor? In addition, our approach follows best practices for balancing comparison groups in longitudinal studies^113^ which we discuss in detail below.

In our study, we apply matching-based methods while considering the SDoH factors as treatments. Prior SDoH research suggests that the five SDoH are interrelated and impact one another^114^. Because of this relationship and known correlations between the SDoH factors, we consider all other SDoH factors as potential confounders of a selected treatment factor. It is true that considering SDoH factors as treatment poses challenges in the framing of the task because these factors are generally not modifiable (e.g., race) or difficult to modify (e.g., income). However, we refer to SDoH factors as treatments, not because they are modifiable, but because we apply the standard formulation of matching-based methods. Identifying modifiable factors in a matching-based experimental study can be used directly to make changes to those treatment factors and to reduce risk. On the other hand, identifying non-modifiable factors has been shown to also be useful to determine high-risk groups that require shielding and targeting for interventions^87^.

Because of the high degrees of spatial segregation in the US^12,101^, matching every ZIP code can be challenging. For example, for every ZIP code with low income and high proportions of Black residents, it is difficult to find a unique ZIP code with high-income and high proportions of Black residents. Therefore, we perform one-to-one matching of ZIP codes with replacement and achieve better matches (i.e., lower bias). Theoretically, this is at the expense of higher variance, but given the size of our dataset, this downside was not a problem in practice. We use the MatchIt package^115^ with the nearest neighbor method and Mahalanobis distance measure to perform the matching.

We leverage an extensive and iterative search across multiple matching methods to achieve maximum covariate balance and representative samples^116^. Regardless of which matching method is superior, one thing to note is that using a better matching method does not generally guarantee a better experimental design. It is then common practice to assess the quality of covariate balance, and in the end, it does not matter how this balance was achieved, as long as it was achieved. We choose to perform matching on all covariates, instead of propensity scoring^117^ which summarizes all of the covariates into one dimension. Importantly, we demonstrate in the Section *Evaluating Quality of Matching Zip Codes* that this method leads to high-quality matches that are balanced across all covariates.

#### Determining treatment and control groups

For each of the SDoH factors, we first split all available ZIP codes into treatment and control groups using a threshold. We use a value close to the median to split the population into two groups for median household income (*$*55,224), % unemployed (3.0%), % with insurance (92.7%), % with internet access (81.8%), and % with Bachelor’s degree or higher (21.1%) because the mean and median of those factors across the ZIP codes are similar. In other cases, the distribution across the ZIP codes is highly skewed. For race/ethnicity, we use the rounded percentage of the national population for that race/ethnicity (12% for Black and 18% for Hispanic residents). For population density, we follow previous practices of urban-rural classification at 500 people per square mile^118^. Supplementary Tables 1 and 2 outline descriptive statistics of our ZIP codes across SDoH factors as well as the national average and our chosen cutoff thresholds.

We consistently defined the treatment group as high-risk according to each of the dimensions of variation we specified^68^. Therefore, our treatment groups are as follows: low income, high percentage of minority residents, low level of educational attainment, high unemployment rate, low insurance rate, low level of internet access, and high population density. For example, for income, we split the ZIP codes into a high-income group (median household income>*$*55,224) and a low-income group (median household income ≤*$*55,224), where the low-income group is the treatment group. Then, for each treatment ZIP code, we look for a control (i.e., low-risk) ZIP code that closely matches it on all other SDoH factors (i.e., ∣*SMD*∣<0.25 to generate a matching pair of ZIP codes). We performed this matching on all ZIP codes, and we discarded ZIP codes for which we cannot find a good match. As demonstrated in Supplementary Table 6, this process retains at least 99.8% of the treatment ZIP codes in our matching process and the discarding of ZIP codes is a rare exception.

#### Evaluating quality of matching zip codes

To gauge whether two ZIP code groups are similar across the SDoH factors and to determine the quality of matching while minimizing the potential confounding effects of these factors, we leverage Standardized Mean Difference (SMD) across ZIP code groups as our measure of comparative quality. The SMD is used to quantify the degree to which two groups are different and is computed by the difference in means of a variable across two groups divided by the standard deviation of the one group (often, the treated group)^111,119,120^. In our analysis, we use ∣*SMD*∣ <0.25 across all our SDoH factors as a criterion to determine that the two groups are comparable, following common practice^109,120^. For example, when we split our ZIP codes in half along median household income to create a high-income ZIP code group (median household income >*$*55,224) and a low-income ZIP code group (median household income ≤*$*55,224) and examine the SMD of other SDoH factors, we find that all SDoH factors except % Hispanic residents and population density fail to achieve the necessary matching criteria of ∣*SMD*∣ < 0.25 prior to matching. This means that low-income ZIP codes are more likely to have less internet access, lower educational attainment level, less health insurance, more unemployment, and higher proportions of Black residents. We perform this evaluation process for all comparison groups to find that correlations among all SDoH factors pose threats to validity in univariate analyses. Supplementary Table 5 summarizes the mean SMD if we were to directly compare two ZIP code groups created by splitting the ZIP codes along the chosen split boundaries. Instead of such direct comparison, we perform matching and tune the caliper of the matching algorithm to determine a good match and to meet the ∣*SMD*∣<0.25 criterion between the two comparison groups across all covariates. Supplementary Table 6 summarizes the result of the matching operation with the maximum ∣*SMD*∣ being below 0.25, that is ensuring comparability across all covariates, between two ZIP code groups along all SDoH factors. Supplementary Tables 7–22 enumerate pre- and post-matching balance assessments between groups for each SDoH factor.

#### Estimating the effect size

After identifying treatment and control ZIP code groups with comparable distributions along all SDoH factors, we compare the outcomes (i.e., constructs of digital engagement such as online access to health condition information) between the matched ZIP code groups. This matching process estimates, for example, the differences in the changes in online health information-seeking behaviors between high and low-income groups during the pandemic while removing plausible contributions from all other observed factors. The differences estimated in this study help identify high-risk groups (e.g., low income, low educational attainment, high proportions of minority residents) for whom to suggest interventions or targeted shielding mitigate or reduce risk^87^.

It is important to note that our matching process only partially incorporates what Helsper calls the digital impact mediators of access, skills, and attitudes^57^. First, where digital access is concerned, though all search queries in the study presume some form of internet access, we do sample ZIP codes with varying levels of aggregate internet access, allowing us to control to some extent for internet access at the population level. It is important to note, however, that our study lacks the data to account for any changes in ZIP code-level internet access during the pandemic due to remote work. Where digital skills are concerned, we do not incorporate direct measures of such technical or operational skills at either the individual or aggregate level, but we do incorporate measures of educational attainment such that we can partially control for this factor in our analysis. Finally, we do not control for individual-level or aggregate-level variation in attitudinal impact mediators such as self-efficacy, as that would be outside the scope of the study. Additional more detailed data would have to be collected and analyzed in order to fully disentangle the impacts of the SDoH factors under study here from such digital impact mediators.

Raw data were collected by proprietary code through Microsoft Bing platform. Study data were extracted from Bing search logs stored on Microsoft’s internal database and processed using its proprietary query language. Data analysis was conducted in Python (v3.9.6) using standard data analysis libraries such as numpy (v1.20.3), scipy (v1.6.2), and pandas (v1.3.1). Visualization was produced using seaborn (v0.11.1). Matching was done using MatchIt (v4.2.0) in R.

### Reporting summary

Further information on research design is available in the Nature Portfolio Reporting Summary linked to this article.

## Supplementary information

Supplementary Information

Reporting Summary

## Data Availability

Raw US census data are publicly available through the Census Reporter API (https://censusreporter.org/). Geographical area measurements are available through the US Census Bureau (https://www.census.gov/geographies/reference-files/2010/geo/state-area.html). Seasonally adjusted US unemployment claims data for 2020 is available through the US Department of Labor (https://oui.doleta.gov/unemploy/claims.asp). The Bing search logs are not publicly available. An aggregated version of the data supporting this study is retained indefinitely for scientific and academic purposes. The data are not publicly available due to privacy and legal restrictions. The data are available on request from the corresponding author with a clear justification and a license agreement. The request will be reviewed and approved case by case by Microsoft Research Release and Compliance team, at which point a license agreement will be drafted and shared.

## Code availability

The code supporting this study is retained indefinitely for scientific and academic purposes. The code is available on request from the corresponding author with a clear justification and a license agreement. The code consists of scripts written in a combination of proprietary query language and standard statistical libraries in R and python. The code will be released alongside data using the process outlined in the Data Availability statement.
