# Supplementary material for: Disparate impacts on online information access during the Covid-19 pandemic
Source: Nat Commun. 2022 Nov 19;13:7094. doi: 10.1038/s41467-022-34592-z (PMC9675823; doi:10.1038/s41467-022-34592-z)
Supplement: Supplementary file 1 — Supplementary Information [file 41467_2022_34592_MOESM1_ESM.pdf]

Supplementary Information

Title

Disparate Impacts on Online Information Access during the COVID-19 Pandemic

Authors

Jina Suh[1,2], Eric Horvitz[1,2], Ryen W. White[1,2], and Tim Althoff[2,*]

[1]Microsoft Research, Redmond, WA, USA
[2]University of Washington, Seattle, WA, USA
[*]althoff@cs.washington.edu

List of Supplementary Information

Supplementary Tables 1 to 22
Supplementary Figures 1 to 26

Supplementary Tables

Supplementary Table 1. Means and medians of eight SDoH factors across 25,801 ZIP codes in our dataset. Populationdensity is computed from the total U.S. population[1] divided by the total U.S. land area[2].

SDoH Factor	ZIP Code Mean	ZIP Code Median	U.S. Mean	ACS Table
Median Household Income	\$60,819.849	\$55,224	\$65,712	B19013
% Unemployed	3.4%	3.0%	2.9%	B23025
% Black or African American Alone	8.3%	1.6%	12.8%	B02001
% Hispanic or Latino	10.1%	4.0%	18.4%	B03003
% with Bachelor or Higher Degree	25.9%	21.1%	33.1%	B15002
Population Density (per sq mile)	1573.966	108.421	92.830*	B01003
% with Health Insurance Coverage	91.1%	92.7%	98.4%	B27001
% with Internet Access	80.5%	81.8%	85.8%	B28003

Supplementary Table 2. Split boundaries used to generate high-risk (treated) and low-risk (control) comparison groups based on ZIP code medians and U.S. national means of eight SDoH factors. Values that informed the choice of the split boundaries are bolded. For population density, we follow previous practices of urban-rural classification[3].

SDoH Factor	ZIP Code Median	U.S. Mean	Boundary	Treated	Control
Median Household Income	**$55,224**	$65,712	$55,224	≤ $55,224	> $55,224
% Unemployed	**3.0%**	2.9%	3.0%	≤ 3.0%	> 3.0%
% Black or African American Alone	1.6%	**12.8%**	12%	≥ 12%	< 12%
% Hispanic or Latino	4.0%	**18.4%**	18%	≥ 18%	< 18%
% with Bachelor or Higher Degree	**21.1%**	33.1%	21.1%	≤ 21.1%	> 21.1%
Population Density (per sq mile)	108.421	92.830	500	≥ 500	< 500
% with Health Insurance Coverage	**92.7%**	98.4%	92.7%	≤ 92.7%	> 92.7%
% with Internet Access	**81.8%**	85.8%	81.8%	≤ 81.8%	> 81.8%

Supplementary Table 3. Pearson correlation coefficients between SDoH factors across 25,801 ZIP codes in our dataset. These correlations reflect socioeconomic segregration and are controlled for through a matching-based approach (Methods). (* = attained Bachelor's degree or higher)

SDoH factor	% Unemp.	% Black	% Hispanic	% BA*	Pop. Density	% Insurance	% Internet
M. H. Income	-0.25	-0.23	-0.07	0.74	0.10	0.41	0.66
% Unemp.		0.32	0.15	-0.18	0.09	-0.23	-0.20
% Black			0.03	-0.09	0.15	-0.19	-0.23
% Hispanic				-0.07	0.21	-0.35	-0.04
% BA*					0.23	0.36	0.62
Pop. Density						0.00	0.10
% Insurance							0.41

Supplementary Table 4. Seven search categories examined and their example search strings and regular expressions. These categories were largely informed by our prior work[4]. In that prior work, we publicly shared the full list of our regular expressions in a github repository (https://github.com/jinasuh/pandemic_needs)

<table>
<tr><th>Search categories</th><th>Example search string</th><th>Example regular expression</th></tr>
<tr><td>Health condition related queries</td><td>arthritis; diabetes</td><td>\b(stroke|diabetes|covid|salmonella|autism|asthma|cancer)\b</td></tr>
<tr><td>Unemployment related queries</td><td>im unemployed; jobless benefits</td><td>\b((i m|im|i am) (unemployed|laid off|furloughed))\b</td></tr>
<tr><td>Clicks to state unemployment sites</td><td>click on www.michigan.gov/uia from search results</td><td>[\/.](www\.michigan\.gov\/uia|www\.in\.gov\/dwd\/2362\.htm)</td></tr>
<tr><td>Financial assistance related queries</td><td>relief fund; financial assistance</td><td>\b((relief|stimulus) (funds?|package|checks?))\b</td></tr>
<tr><td>Clicks to online learning sites</td><td>click on coursera.org from search results</td><td>[\/.](quizlet\.com|mooc-list\.com|coursera\.org|khanacademy\.org)</td></tr>
<tr><td>Food delivery related queries</td><td>grocery delivery; deliver meal</td><td>\b((food|grocery|meal) delivery)\b</td></tr>
<tr><td>Food assistance related queries</td><td>food stamps; snap program</td><td>b(food assistance|snap program|food stamps|food bank|food pantry)\b</td></tr>
</table>

Supplementary Table 5. Summary of *pre-matching* balance assessment and sample sizes across SDoH factors. $|SMD| > 0.25$ indicates that the two ZIP code groups created along the split boundary are different and, thus, not comparable.

SDoH Factor	Split Boundary	Treated Count	Control Count	Max SMD	Mean SMD
Median Household Income	$55,224	12637	12637	1.018	0.459
% Unemployed	3.0%	12637	12637	0.385	0.269
% Black or Afr. Am. Alone	12%	4945	20329	0.467	0.300
% Hispanic or Latino	18%	4028	21246	0.591	0.218
% with Bachelor or Higher	21.1%	12637	12637	0.975	0.416
Population Density (per mi^2)	500	8037	17237	0.689	0.392
% with Health Ins. Cov.	92.7%	12666	12608	0.629	0.396
% with Internet Access	81.8%	12637	12637	0.974	0.429

Supplementary Table 6. Summary of *post-matching* balance assessment and sample sizes across SDoH factors fortreated and control groups.

SDoH Factor	Max SMD	Mean SMD	Tr. Matched	Tr. Unmat.	Ct. Matched	Ct. Unmat.
Median Household Income	0.236	0.105	12555	21	3854	8720
% Unemployed	0.088	0.026	12544	31	5917	6658
% Black or Afr. Am. Alone	0.144	0.072	4932	0	1603	18615
% Hispanic or Latino	0.131	0.082	4018	0	2247	18885
% with Bachelor or Higher	0.113	0.069	12572	3	3981	8594
Population Density (per mi^2)	0.181	0.114	8022	6	2836	14286
% with Health Ins. Cov.	0.067	0.030	8508	0	4339	12303
% with Internet Access	0.176	0.075	12575	0	3623	8952

Supplementary Table 7. Balance assessment between unmatched high (Treated) and low (Control) '% Black or African American Alone' Groups

SDoH Factor	Means Treated	Means Control	Mean Diff.	Std. Mean Diff.
distance	0.590	0.100	0.490	1.606
Median Household Income	49810.138	63497.945	-13687.808	-0.680
Unemployed	0.045	0.031	0.015	0.628
Hispanic or Latino	0.125	0.095	0.030	0.201
With Bachelor or Higher Degree Attained	0.232	0.265	-0.034	-0.244
Population Density (per sq mile)	2975.905	1172.126	1803.778	0.245
With Health Insurance Coverage	0.883	0.918	-0.034	-0.575
With Internet Access	0.766	0.816	-0.050	-0.414
Black or African American Alone	0.337	0.021	0.316	1.517

Supplementary Table 8. Balance assessment between matched high (Treated) and low (Control) '% Black or African American Alone' groups

SDoH Factor	Means Treated	Means Control	Mean Diff.	Std. Mean Diff.
distance	0.887	0.791	0.096	0.043
Median Household Income	49835.515	52509.535	-2674.020	-0.133
Unemployed	0.045	0.043	0.002	0.097
Hispanic or Latino	0.125	0.126	-0.001	-0.004
With Bachelor or Higher Degree Attained	0.232	0.240	-0.008	-0.059
Population Density (per sq mile)	2981.898	2250.314	731.584	0.099
With Health Insurance Coverage	0.884	0.881	0.003	0.044
With Internet Access	0.766	0.775	-0.009	-0.072
Black or African American Alone	0.337	0.041	0.297	1.425

Supplementary Table 9. Balance assessment between unmatched high (Treated) and low (Control) '% Hispanic or Latino' Groups

SDoH Factor	Means Treated	Means Control	Mean Diff.	Std. Mean Diff.
distance	0.368	0.120	0.249	1.023
Median Household Income	57217.731	61502.769	-4285.038	-0.209
Unemployed	0.041	0.032	0.009	0.398
Black or African American Alone	0.099	0.080	0.019	0.145
With Bachelor or Higher Degree Attained	0.234	0.264	-0.030	-0.224
Population Density (per sq mile)	4021.757	1051.698	2970.060	0.333
With Health Insurance Coverage	0.864	0.920	-0.056	-0.751
With Internet Access	0.806	0.806	-0.000	-0.001
Hispanic or Latino	0.399	0.044	0.355	1.736

Supplementary Table 10. Balance assessment between matched high (Treated) and low (Control) '% Hispanic or Latino' groups

SDoH Factor	Means Treated	Means Control	Mean Diff.	Std. Mean Diff.
distance	-0.639	-0.699	0.060	0.042
Median Household Income	57234.061	58095.893	-861.832	-0.042
Unemployed	0.041	0.038	0.002	0.105
Black or African American Alone	0.099	0.085	0.014	0.111
With Bachelor or Higher Degree Attained	0.234	0.239	-0.006	-0.042
Population Density (per sq mile)	4029.507	2986.171	1043.336	0.117
With Health Insurance Coverage	0.864	0.865	-0.001	-0.018
With Internet Access	0.806	0.817	-0.010	-0.096
Hispanic or Latino	0.399	0.086	0.313	1.530

Supplementary Table 11. Balance assessment between unmatched high (Control) and low (Treated) 'Median Household Income' Groups

SDoH Factor	Means Treated	Means Control	Mean Diff.	Std. Mean Diff.
distance	0.725	0.275	0.450	1.855
Unemployed	0.039	0.028	0.011	0.412
Black or African American Alone	0.117	0.049	0.068	0.343
Hispanic or Latino	0.110	0.091	0.019	0.102
With Bachelor or Higher Degree Attained	0.177	0.341	-0.164	-1.751
Population Density (per sq mile)	1266.027	1784.065	-518.038	-0.107
With Health Insurance Coverage	0.887	0.934	-0.047	-0.692
With Internet Access	0.744	0.868	-0.125	-1.257
Median Household Income	43356.749	78282.949	-34926.200	-4.189

Supplementary Table 12. Balance assessment between matched high (Control) and low (Treated) 'Median Household Income' groups

SDoH Factor	Means Treated	Means Control	Mean Diff.	Std. Mean Diff.
distance	1.567	1.449	0.119	0.060
Unemployed	0.039	0.036	0.003	0.113
Black or African American Alone	0.117	0.092	0.025	0.126
Hispanic or Latino	0.111	0.091	0.020	0.107
With Bachelor or Higher Degree Attained	0.177	0.180	-0.003	-0.029
Population Density (per sq mile)	1272.878	747.235	525.643	0.108
With Health Insurance Coverage	0.888	0.889	-0.001	-0.021
With Internet Access	0.745	0.746	-0.001	-0.011
Median Household Income	43401.084	62615.121	-19214.037	-2.306

Supplementary Table 13. Balance assessment between unmatched high (Control) and low (Treated) '% with Bachelor or Higher Degree Attained' Groups

SDoH Factor	Means Treated	Means Control	Mean Diff.	Std. Mean Diff.
distance	0.714	0.286	0.429	1.869
Median Household Income	47879.339	73760.359	-25881.020	-2.046
Unemployed	0.037	0.029	0.008	0.308
Black or African American Alone	0.095	0.072	0.023	0.125
Hispanic or Latino	0.109	0.093	0.016	0.086
Population Density (per sq mile)	788.145	2261.947	-1473.803	-0.441
With Health Insurance Coverage	0.890	0.931	-0.041	-0.571
With Internet Access	0.745	0.867	-0.121	-1.222
With Bachelor or Higher Degree Attained	0.141	0.376	-0.235	-5.334

Supplementary Table 14. Balance assessment between matched high (Control) and low (Treated) '% with Bachelor or Higher Degree Attained' groups

SDoH Factor	Means Treated	Means Control	Mean Diff.	Std. Mean Diff.
distance	1.336	1.242	0.094	0.058
Median Household Income	47895.952	48454.763	-558.811	-0.044
Unemployed	0.037	0.036	0.002	0.073
Black or African American Alone	0.095	0.084	0.011	0.060
Hispanic or Latino	0.109	0.097	0.013	0.066
Population Density (per sq mile)	793.735	751.143	42.592	0.013
With Health Insurance Coverage	0.891	0.897	-0.007	-0.097
With Internet Access	0.746	0.751	-0.005	-0.050
With Bachelor or Higher Degree Attained	0.142	0.269	-0.128	-2.903

Supplementary Table 15. Balance assessment between unmatched high (Treated) and low (Control) 'Population Density (per sq mile)' Groups

SDoH Factor	Means Treated	Means Control	Mean Diff.	Std. Mean Diff.
distance	0.645	0.166	0.479	1.701
Median Household Income	71129.001	56013.058	15115.943	0.462
Unemployed	0.038	0.031	0.006	0.322
Black or African American Alone	0.144	0.055	0.089	0.450
Hispanic or Latino	0.169	0.069	0.100	0.522
With Bachelor or Higher Degree Attained	0.364	0.210	0.154	0.805
With Health Insurance Coverage	0.915	0.909	0.006	0.097
With Internet Access	0.860	0.781	0.079	0.877
Population Density (per sq mile)	4599.740	91.425	4508.315	0.510

Supplementary Table 16. Balance assessment between matched high (Treated) and low (Control) 'Population Density (per sq mile)' groups

SDoH Factor	Means Treated	Means Control	Mean Diff.	Std. Mean Diff.
distance	0.882	0.763	0.118	0.067
Median Household Income	71169.714	72899.376	-1729.662	-0.053
Unemployed	0.038	0.035	0.002	0.132
Black or African American Alone	0.144	0.124	0.020	0.099
Hispanic or Latino	0.169	0.154	0.015	0.079
With Bachelor or Higher Degree Attained	0.363	0.371	-0.007	-0.038
With Health Insurance Coverage	0.915	0.925	-0.010	-0.156
With Internet Access	0.860	0.873	-0.013	-0.147
Population Density (per sq mile)	4598.958	227.548	4371.410	0.494

Supplementary Table 17. Balance assessment between unmatched high (Treated) and low (Control) '% Unemployed' Groups

SDoH Factor	Means Treated	Means Control	Mean Diff.	Std. Mean Diff.
distance	0.581	0.419	0.162	0.781
Median Household Income	55178.441	66461.257	-11282.816	-0.503
Black or African American Alone	0.125	0.042	0.083	0.421
Hispanic or Latino	0.132	0.070	0.062	0.328
With Bachelor or Higher Degree Attained	0.230	0.288	-0.058	-0.416
Population Density (per sq mile)	2062.020	988.072	1073.948	0.173
With Health Insurance Coverage	0.898	0.924	-0.026	-0.404
With Internet Access	0.791	0.821	-0.029	-0.272
Unemployed	0.049	0.018	0.030	1.401

Supplementary Table 18. Balance assessment between matched high (Treated) and low (Control) '% Unemployed'groups

SDoH Factor	Means Treated	Means Control	Mean Diff.	Std. Mean Diff.
distance	0.460	0.423	0.037	0.032
Median Household Income	55192.003	55360.831	-168.828	-0.008
Black or African American Alone	0.124	0.122	0.002	0.010
Hispanic or Latino	0.131	0.125	0.006	0.030
With Bachelor or Higher Degree Attained	0.230	0.225	0.004	0.030
Population Density (per sq mile)	1902.049	1476.827	425.222	0.069
With Health Insurance Coverage	0.898	0.898	-0.001	-0.010
With Internet Access	0.791	0.791	0.001	0.006
Unemployed	0.048	0.021	0.028	1.303

Supplementary Table 19. Balance assessment between unmatched high (Control) and low (Treated) '% with Health Insurance Coverage' Groups

SDoH Factor	Means Treated	Means Control	Mean Diff.	Std. Mean Diff.
distance	0.528	0.242	0.286	1.140
Median Household Income	48183.387	67291.556	-19108.168	-1.336
Unemployed	0.040	0.030	0.010	0.374
Black or African American Alone	0.133	0.058	0.075	0.378
Hispanic or Latino	0.167	0.067	0.101	0.458
With Bachelor or Higher Degree Attained	0.187	0.296	-0.109	-1.076
Population Density (per sq mile)	1529.525	1522.752	6.774	0.001
With Internet Access	0.752	0.834	-0.082	-0.737
With Health Insurance Coverage	0.841	0.947	-0.106	-1.770

Supplementary Table 20. Balance assessment between matched high (Control) and low (Treated) '% with Health Insurance Coverage' groups

SDoH Factor	Means Treated	Means Control	Mean Diff.	Std. Mean Diff.
distance	0.255	0.199	0.055	0.038
Median Household Income	48177.005	48170.216	6.789	0.000
Unemployed	0.040	0.039	0.001	0.025
Black or African American Alone	0.133	0.126	0.007	0.035
Hispanic or Latino	0.168	0.158	0.009	0.043
With Bachelor or Higher Degree Attained	0.187	0.187	0.000	0.005
Population Density (per sq mile)	1537.624	1250.643	286.982	0.060
With Internet Access	0.752	0.753	-0.001	-0.008
With Health Insurance Coverage	0.841	0.932	-0.092	-1.530

Supplementary Table 21. Balance assessment between unmatched high (Control) and low (Treated) '% with Internet Access' Groups

SDoH Factor	Means Treated	Means Control	Mean Diff.	Std. Mean Diff.
distance	0.749	0.251	0.497	2.260
Median Household Income	46716.098	74923.599	-28207.501	-2.274
Unemployed	0.037	0.030	0.007	0.256
Black or African American Alone	0.107	0.060	0.047	0.244
Hispanic or Latino	0.102	0.099	0.003	0.019
With Bachelor or Higher Degree Attained	0.168	0.350	-0.182	-2.326
Population Density (per sq mile)	1003.589	2046.502	-1042.913	-0.225
With Health Insurance Coverage	0.889	0.932	-0.043	-0.603
With Internet Access	0.723	0.889	-0.166	-1.980

Supplementary Table 22. Balance assessment between matched high (Control) and low (Treated) '% with Internet Access' groups

SDoH Factor	Means Treated	Means Control	Mean Diff.	Std. Mean Diff.
distance	1.436	1.234	0.202	0.136
Median Household Income	46721.584	48710.356	-1988.773	-0.160
Unemployed	0.037	0.035	0.001	0.056
Black or African American Alone	0.107	0.096	0.011	0.059
Hispanic or Latino	0.103	0.093	0.009	0.050
With Bachelor or Higher Degree Attained	0.168	0.173	-0.005	-0.066
Population Density (per sq mile)	1009.178	898.001	111.178	0.024
With Health Insurance Coverage	0.889	0.893	-0.003	-0.049
With Internet Access	0.724	0.854	-0.130	-1.559

Supplementary Figures

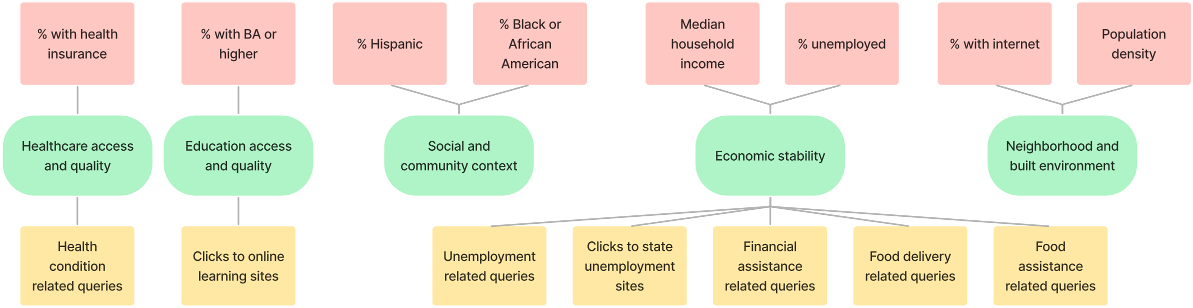

Supplementary Figure 1. Our choice of census variables (red) and search categories (yellow) are inspired by the Social Determinants of Health framework (green).

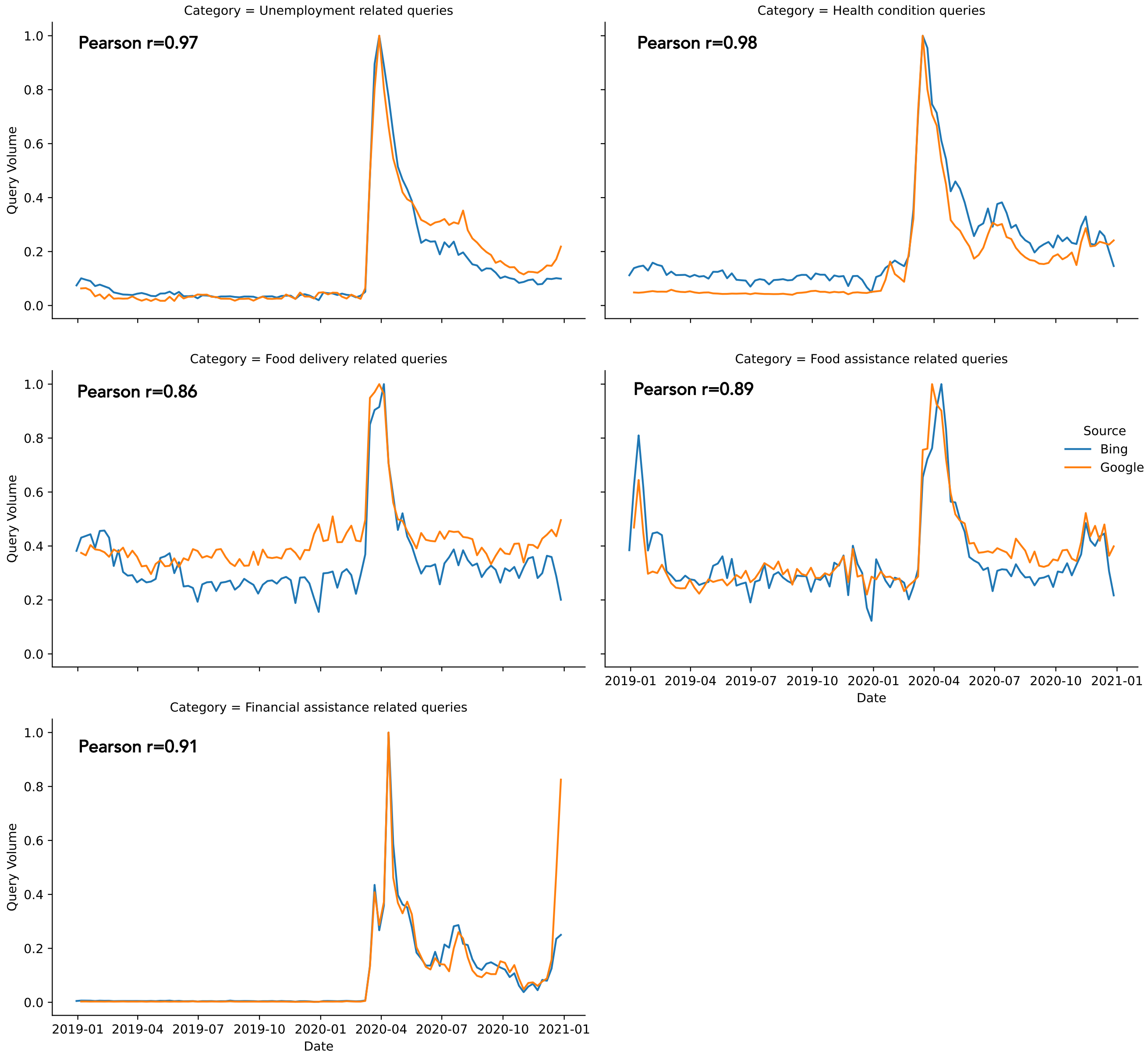

Supplementary Figure 2. Normalized query volumes across five categories in years 2019 and 2020 across Bing and Google.

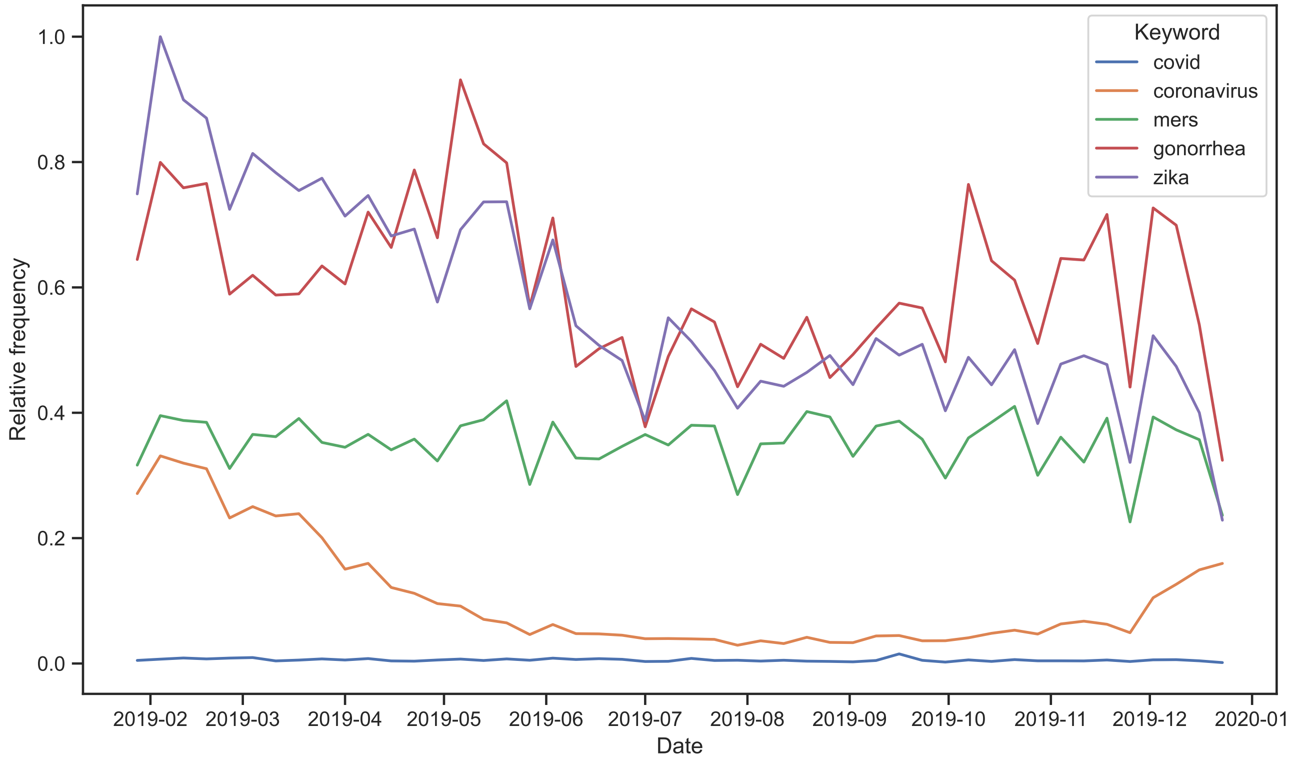

Supplementary Figure 3. Frequency of search keywords “covid”, “coronavirus”, “mers”, “gonorrhea”, and “zika” in 2019, scaled to the maximum frequency within the data presented in the figure.

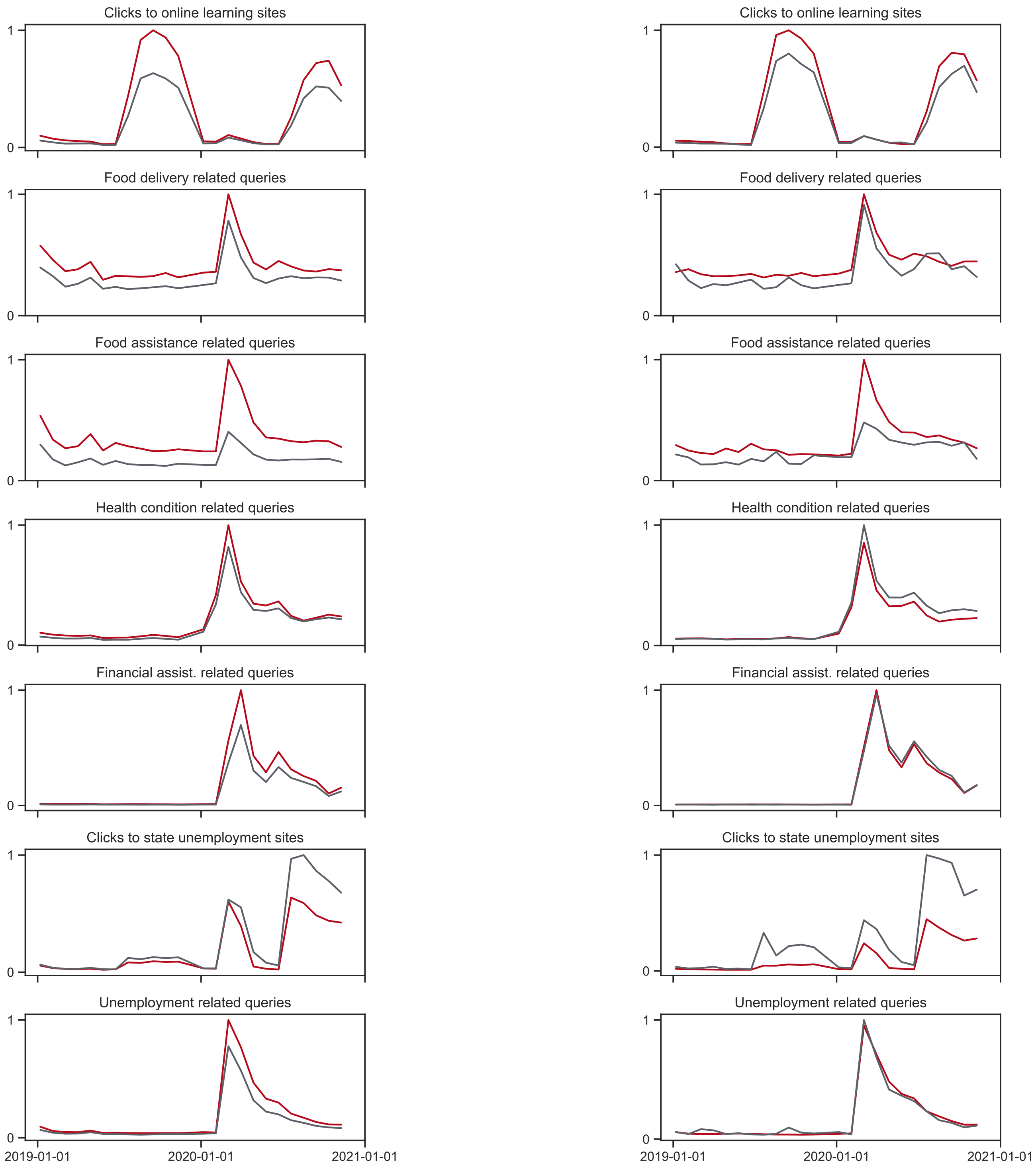

(a) Average query frequencies per ZIP code

(b) Average query proportions per ZIP code

Supplementary Figure 4. (a) Average query frequencies per ZIP code across seven search categories, and (b) average proportion of total queries per ZIP code across seven search categories. Query frequencies and proportions are scaled to the maximum of 1 within each figure. Red lines indicate ZIP codes with % of the population with Black or African American alone ≥ 12%, and gray lines indicate ZIP codes with % of the population with Black or African American alone < 12%.

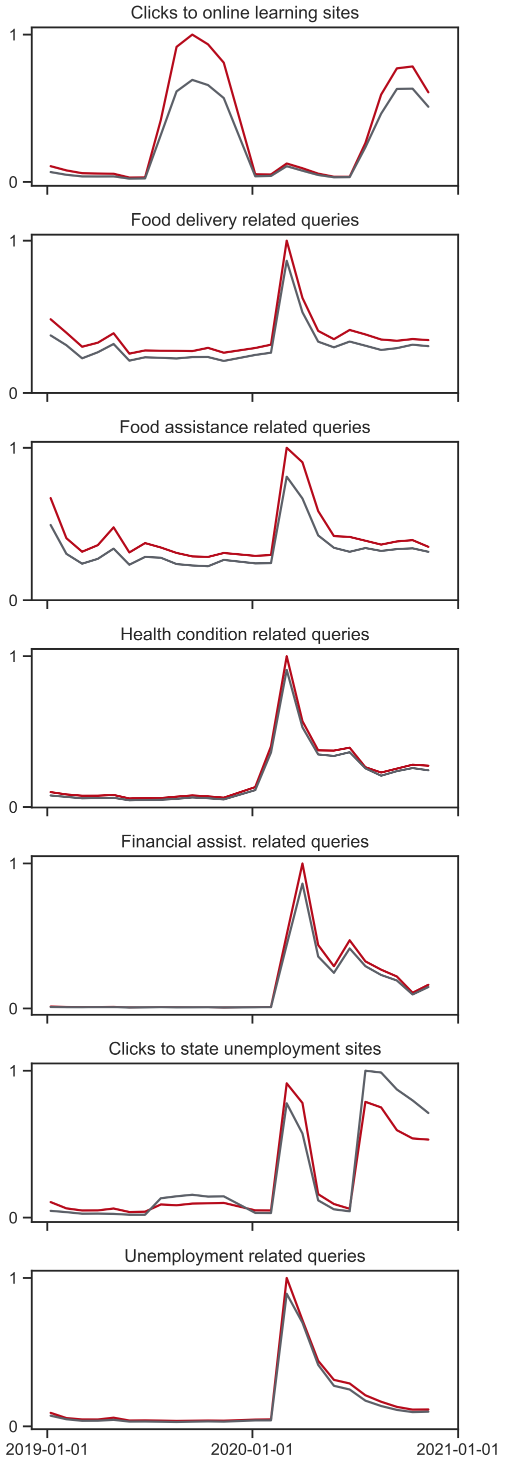

(a) Average query frequencies per ZIP code

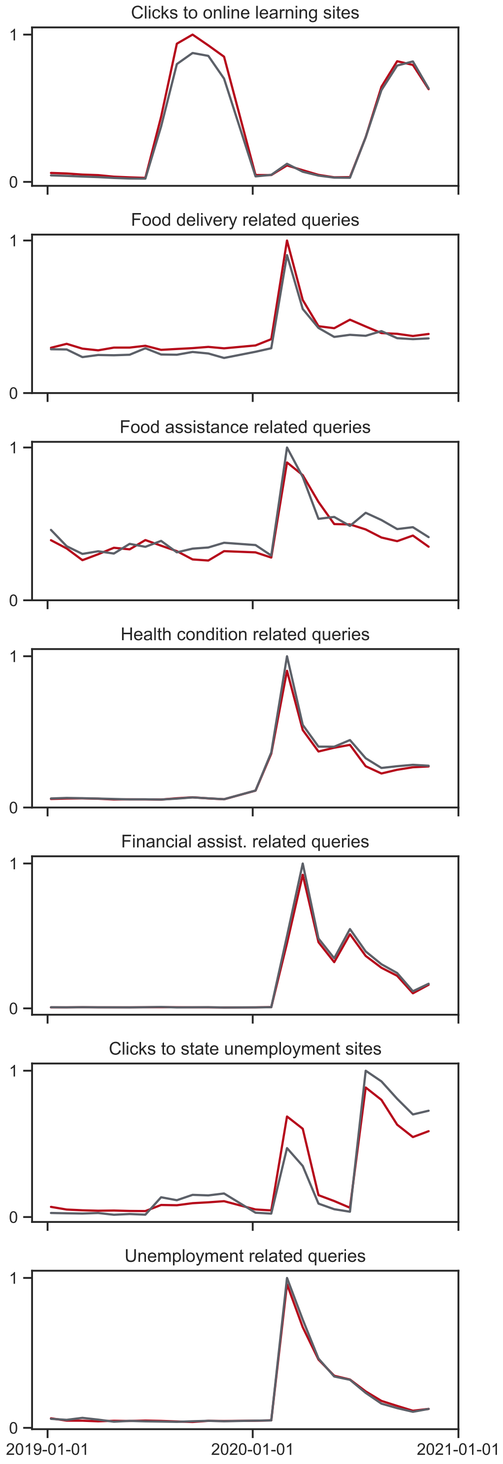

(b) Average query proportions per ZIP code

Supplementary Figure 5. (a) Average query frequencies per ZIP code across seven search categories, and (b) average proportion of total queries per ZIP code across seven search categories. Query frequencies and proportions are scaled to the maximum of 1 within each figure. Red lines indicate ZIP codes with % of the population with Hispanic origin $\geq$18%, and gray lines indicate ZIP codes with % of the population with Hispanic origin $<$ 18%.

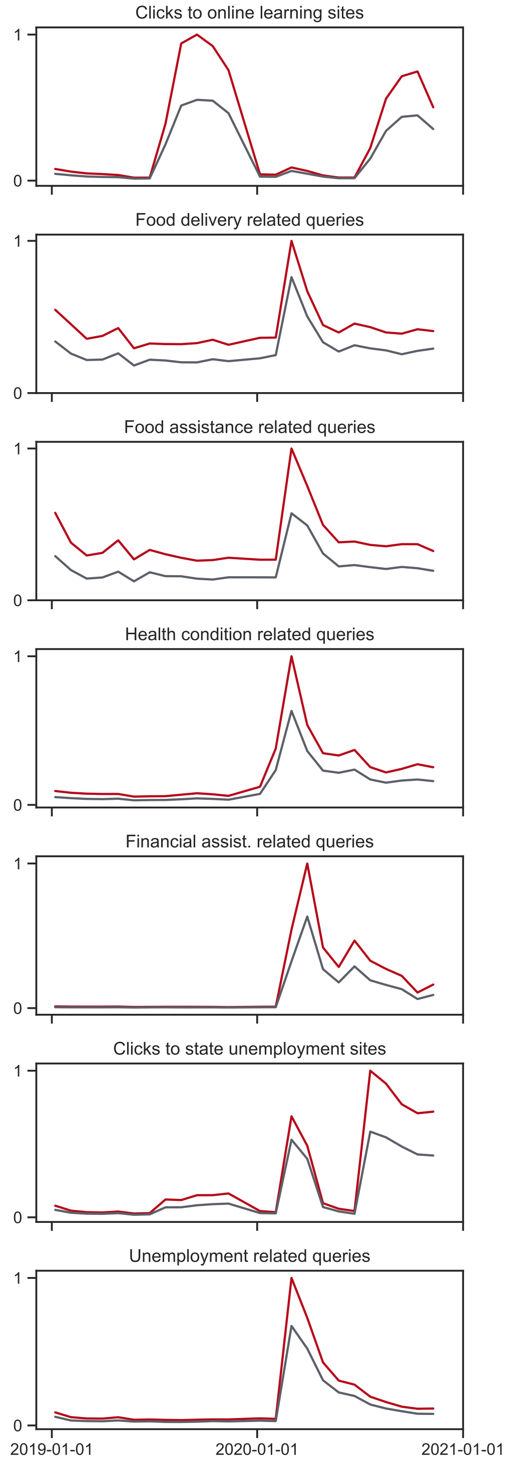

(a) Average query frequencies per ZIP code

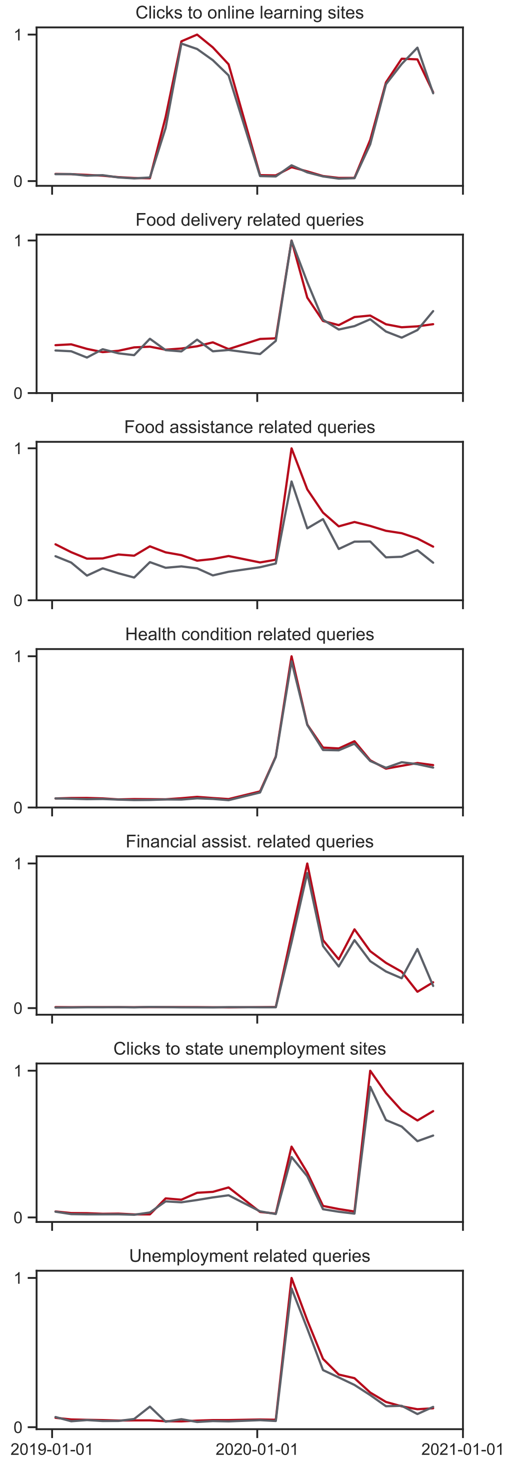

(b) Average query proportions per ZIP code

Supplementary Figure 6. (a) Average query frequencies per ZIP code across seven search categories, and (b) average proportion of total queries per ZIP code across seven search categories. Query frequencies and proportions are scaled to the maximum of 1 within each figure. Red lines indicate ZIP codes with median household income $\leq$ \$55,224, and gray lines indicate ZIP codes with median household income > \$55,224.

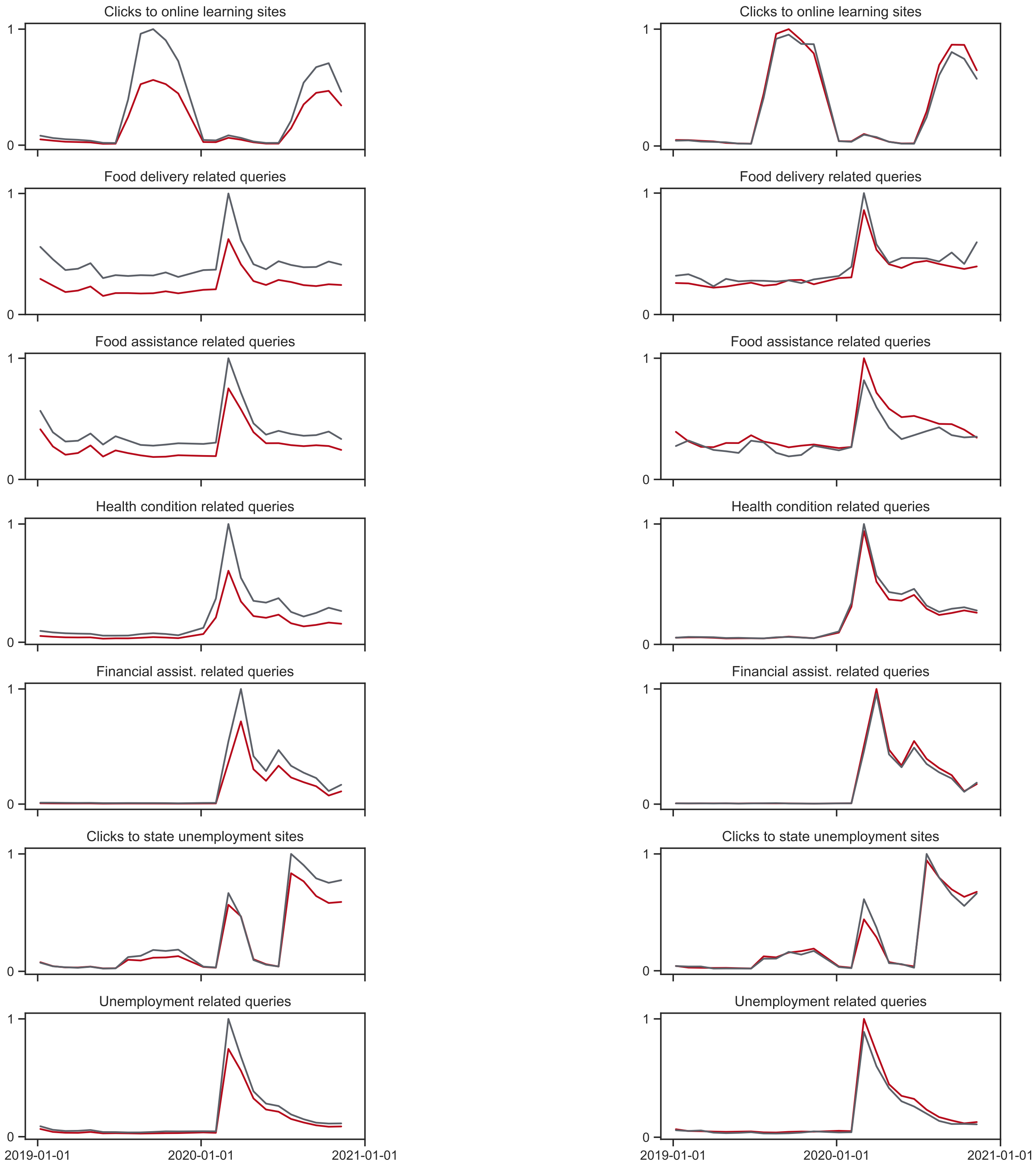

(a) Average query frequencies per ZIP code

(b) Average query proportions per ZIP code

Supplementary Figure 7. (a) Average query frequencies per ZIP code across seven search categories, and (b) average proportion of total queries per ZIP code across seven search categories. Query frequencies and proportions are scaled to the maximum of 1 within each figure. Red lines indicate ZIP codes with % of the population with Bachelor's degree or higher degrees $\leq 21\%$, and gray lines indicate ZIP codes with Bachelor's degree or higher degrees $> 21\%$.

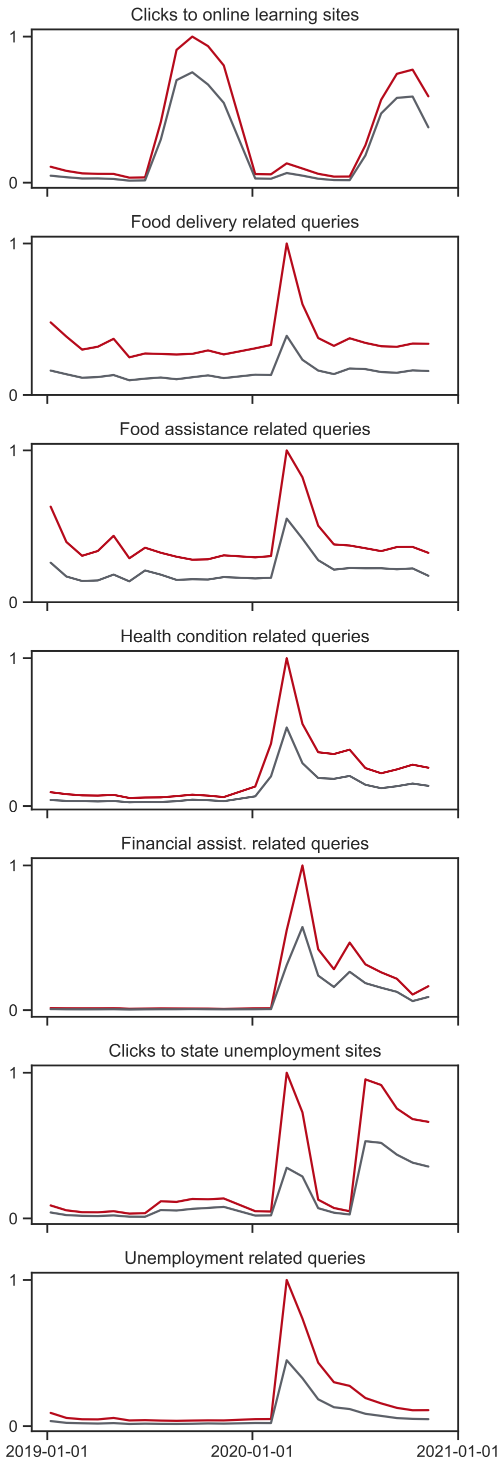

(a) Average query frequencies per ZIP code

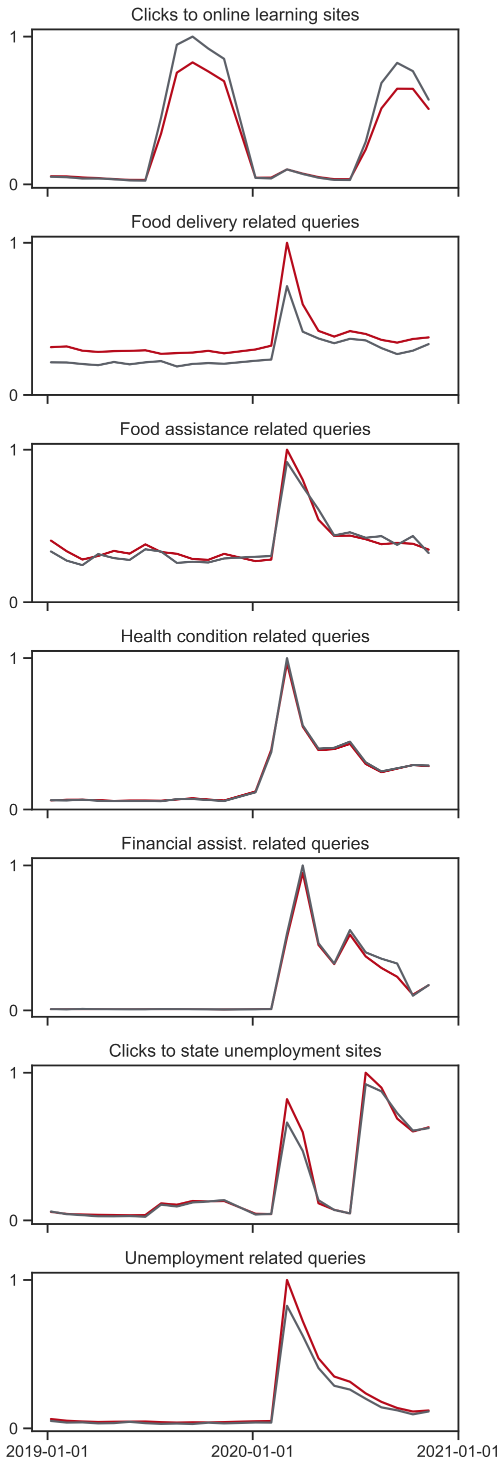

(b) Average query proportions per ZIP code

Supplementary Figure 8. (a) Average query frequencies per ZIP code across seven search categories, and (b) average proportion of total queries per ZIP code across seven search categories. Query frequencies and proportions are scaled to the maximum of 1 within each figure. Red lines indicate ZIP codes with population density ≥ 500 people per square mile, and gray lines indicate ZIP codes with internet access < 500 people per square mile.

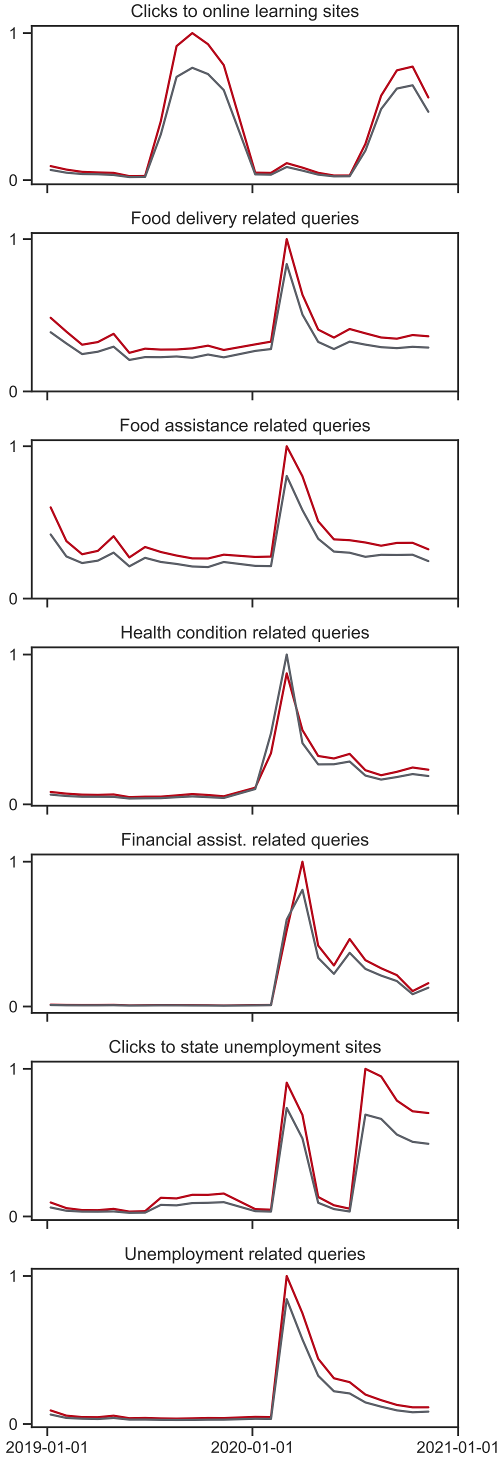

(a) Average query frequencies per ZIP code

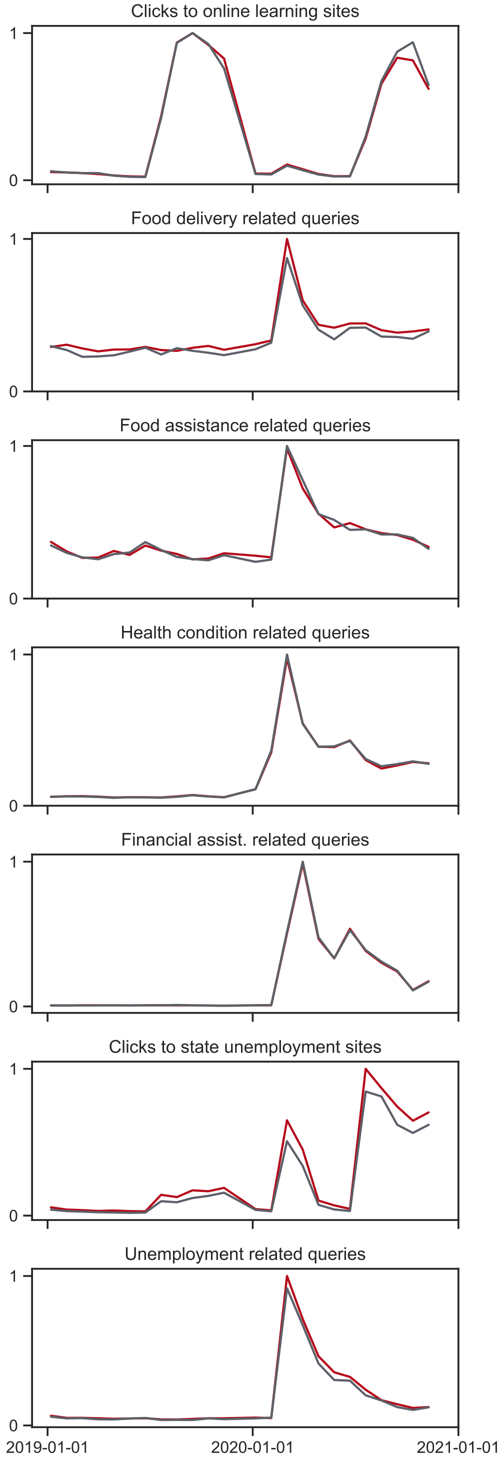

(b) Average query proportions per ZIP code

Supplementary Figure 9. (a) Average query frequencies per ZIP code across seven search categories, and (b) average proportion of total queries per ZIP code across seven search categories. Query frequencies and proportions are scaled to the maximum of 1 within each figure. Red lines indicate ZIP codes with % of the population unemployed $\geq 3\%$, and gray lines indicate ZIP codes with % of the population unemployed $< 3\%$.

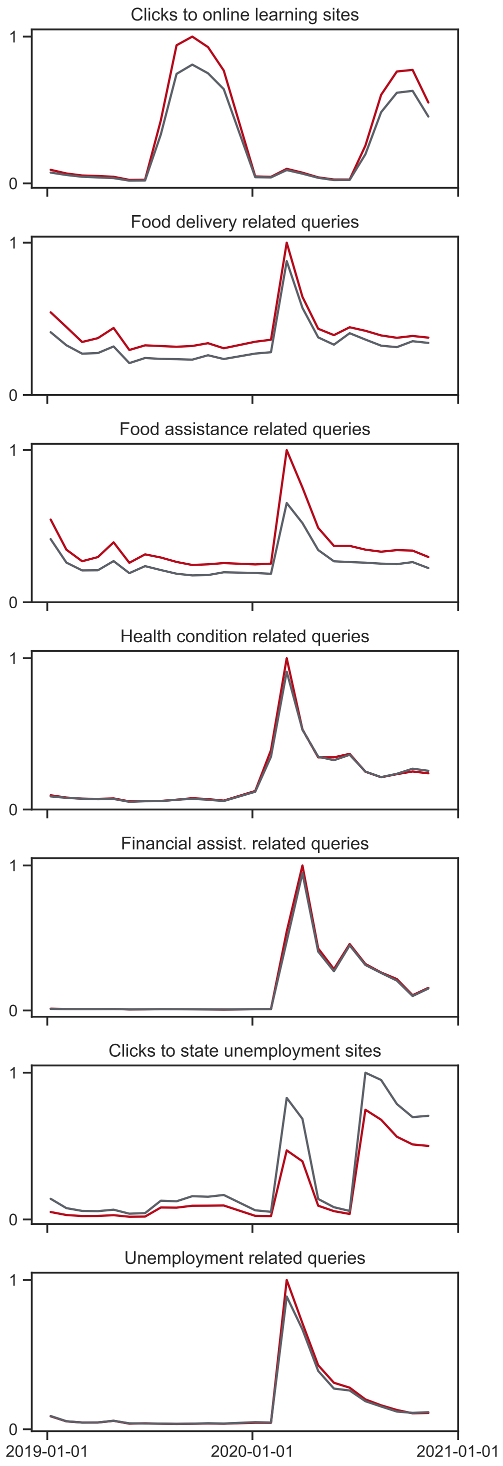

(a) Average query frequencies per ZIP code

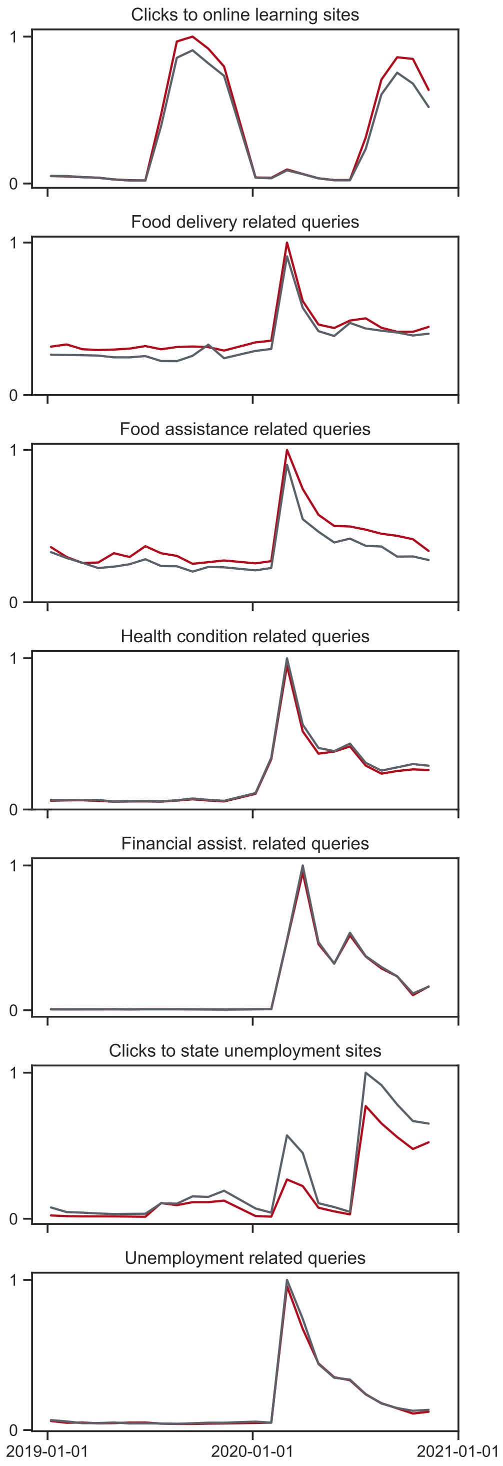

(b) Average query proportions per ZIP code

Supplementary Figure 10. (a) Average query frequencies per ZIP code across seven search categories, and (b) average proportion of total queries per ZIP code across seven search categories. Query frequencies and proportions are scaled to the maximum of 1 within each figure. Red lines indicate ZIP codes with % of the population with health insurance coverage $\leq 93\%$, and gray lines indicate ZIP codes with health insurance coverage $> 93\%$.

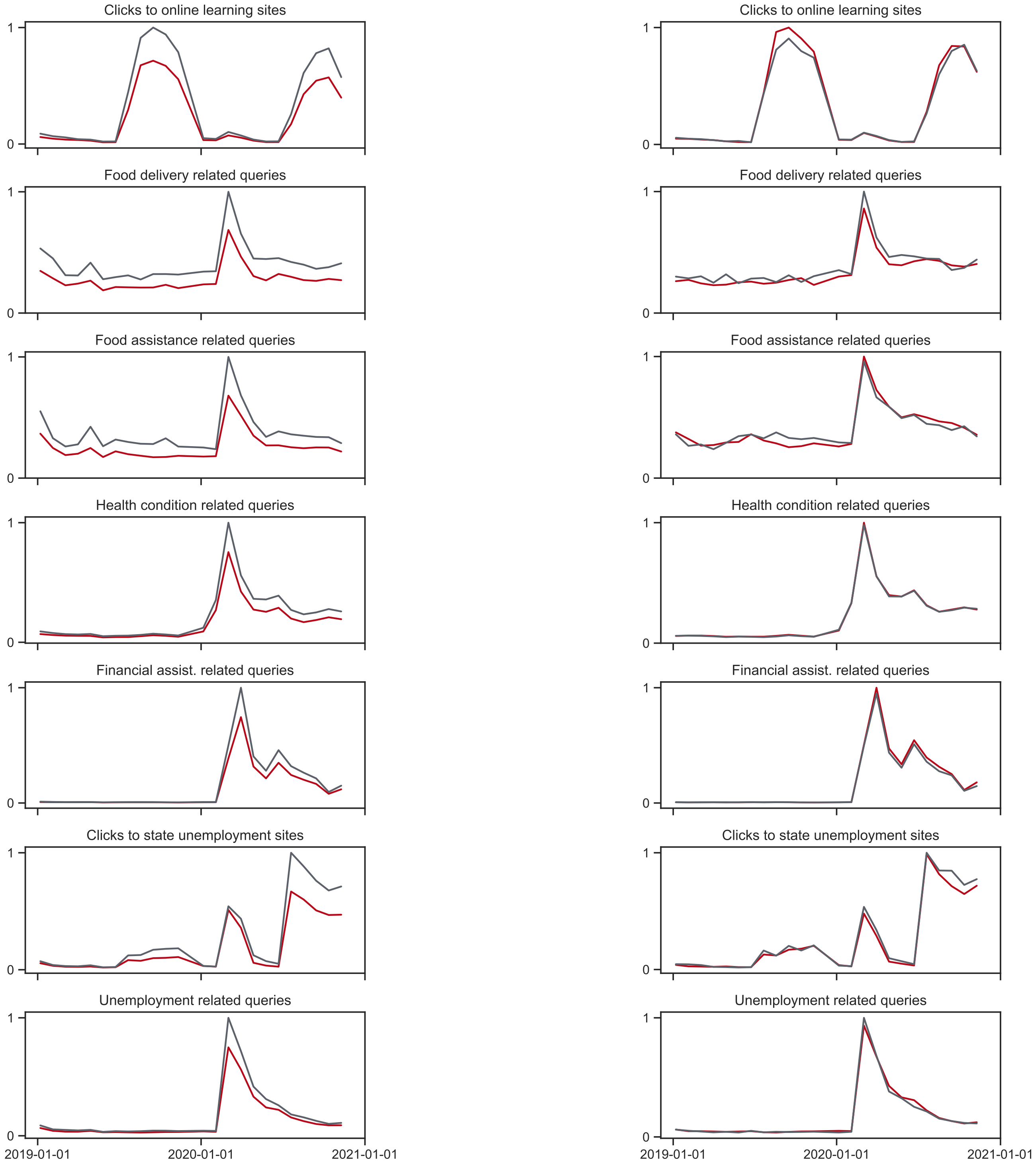

(a) Average query frequencies per ZIP code

(b) Average query proportions per ZIP code

Supplementary Figure 11. (a) Average query frequencies per ZIP code across seven search categories, and (b) average proportion of total queries per ZIP code across seven search categories. Query frequencies and proportions are scaled to the maximum of 1 within each figure. Red lines indicate ZIP codes with % of the population with internet access ≤82%, and gray lines indicate ZIP codes with internet access > 82%.

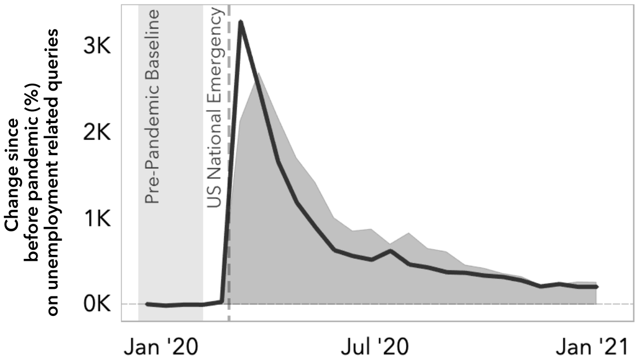

(a) Claims vs unemployment related queries

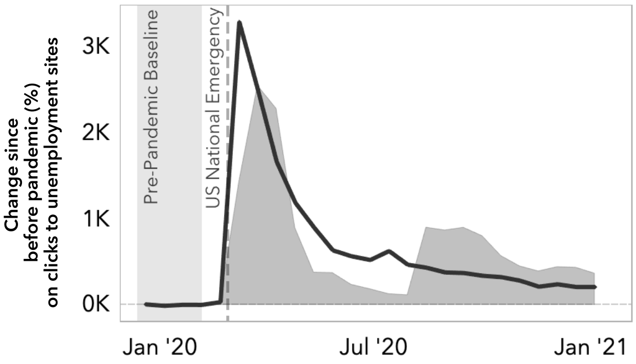

(b) Claims vs clicks to unemployment sites

Supplementary Figure 12. (a) Percent change in the unemployment related queries in Bing (shaded) and the reported unemployment claims from the US Department of Labor (line,https://oui.doleta.gov/unemploy/claims.asp) compared to pre-pandemic baseline. (b) Percent change in the clicks to unemployment sites in Bing (shaded) and the reported unemployment claims from the US Department of Labor (line, https://oui.doleta.gov/unemploy/claims.asp) compared to pre-pandemic baseline.

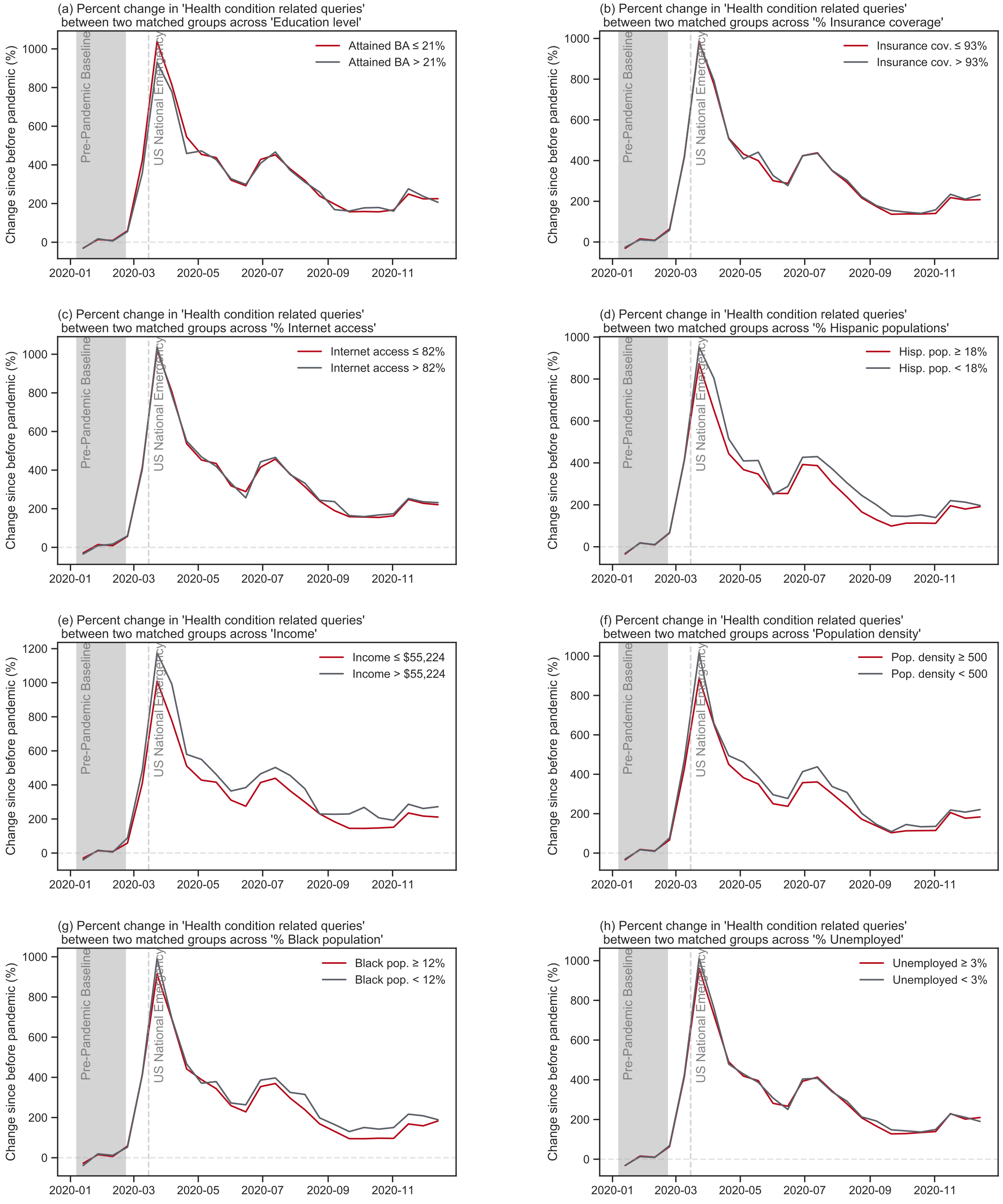

Supplementary Figure 13. Percent change in ‘Health condition related queries’ between two matched groups across eight SDoHfactors.

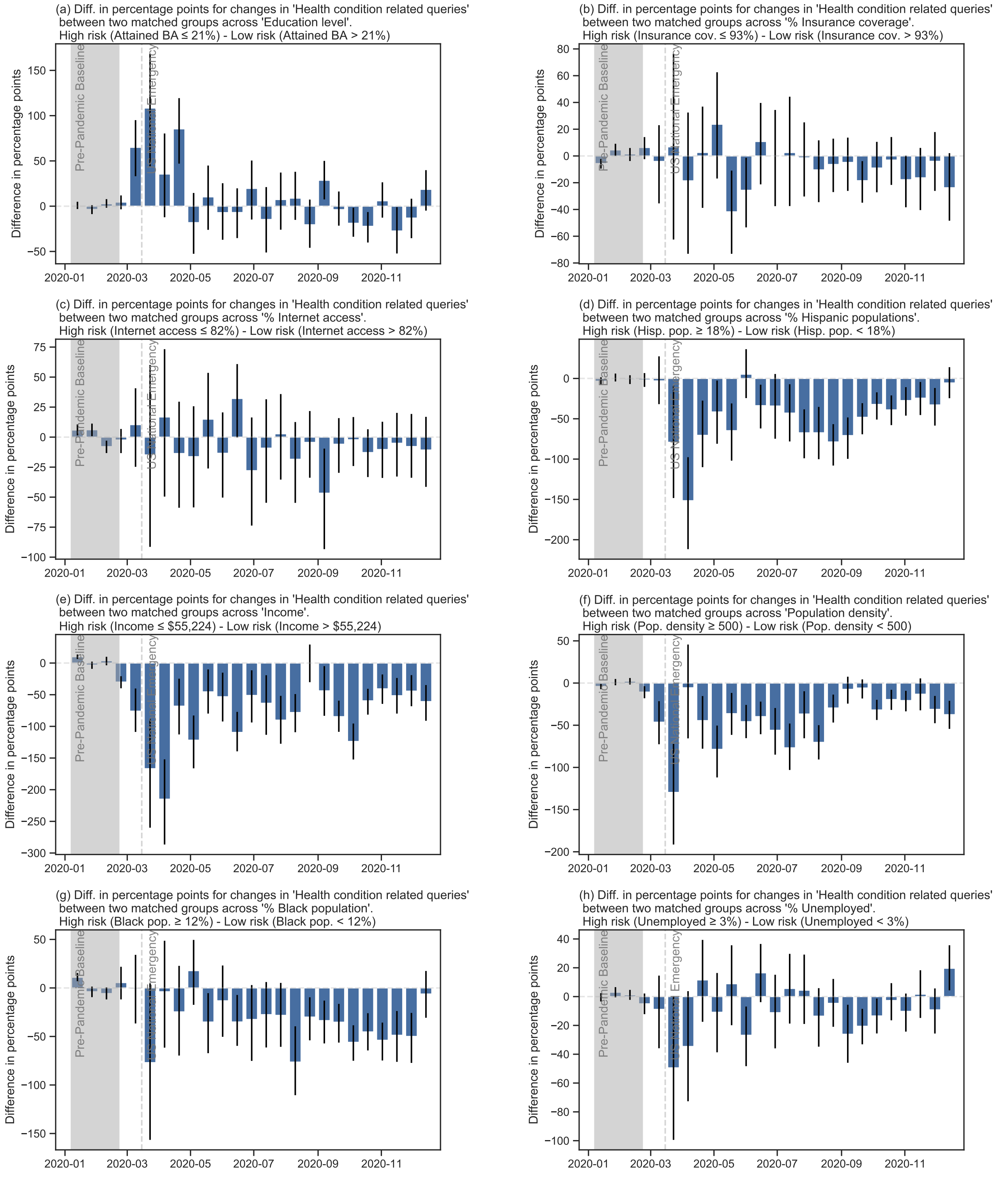

Supplementary Figure 14. Differences in percentage points for changes in ‘Health condition related queries’ between two matched groups across eight SDoH factors. Data are presented as mean values, and error bars indicate 95% confidence intervals ($1{,}603 \leq n \leq 12{,}575$, see Supplementary Table 6 for sample sizes per SDoH factor). Both mean values and confidence intervals are obtained through bootstrapping with 500 iterations.

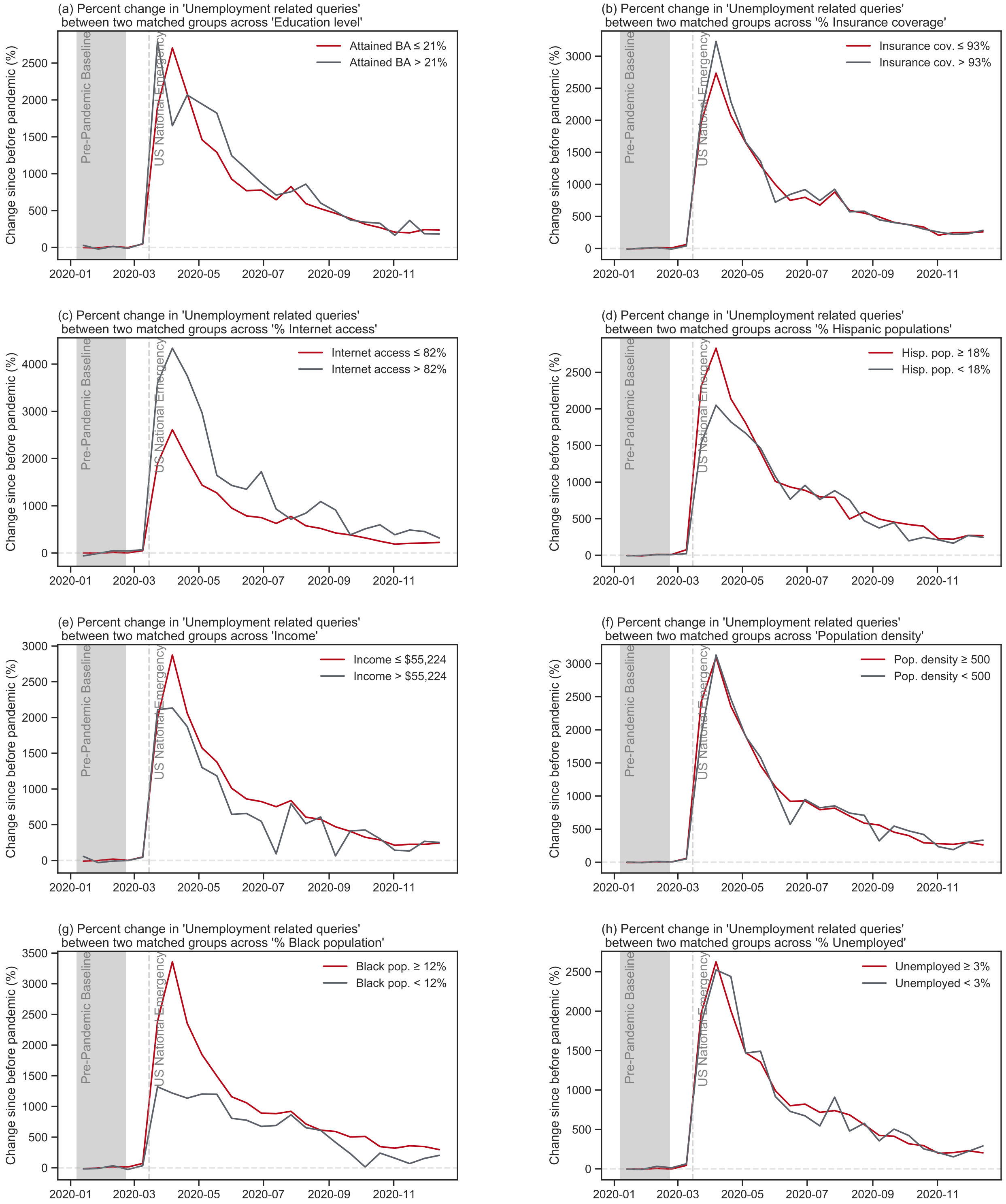

Supplementary Figure 15. Percent change in 'Unemployment related queries' between two matched groups across eight SDoHfactors.

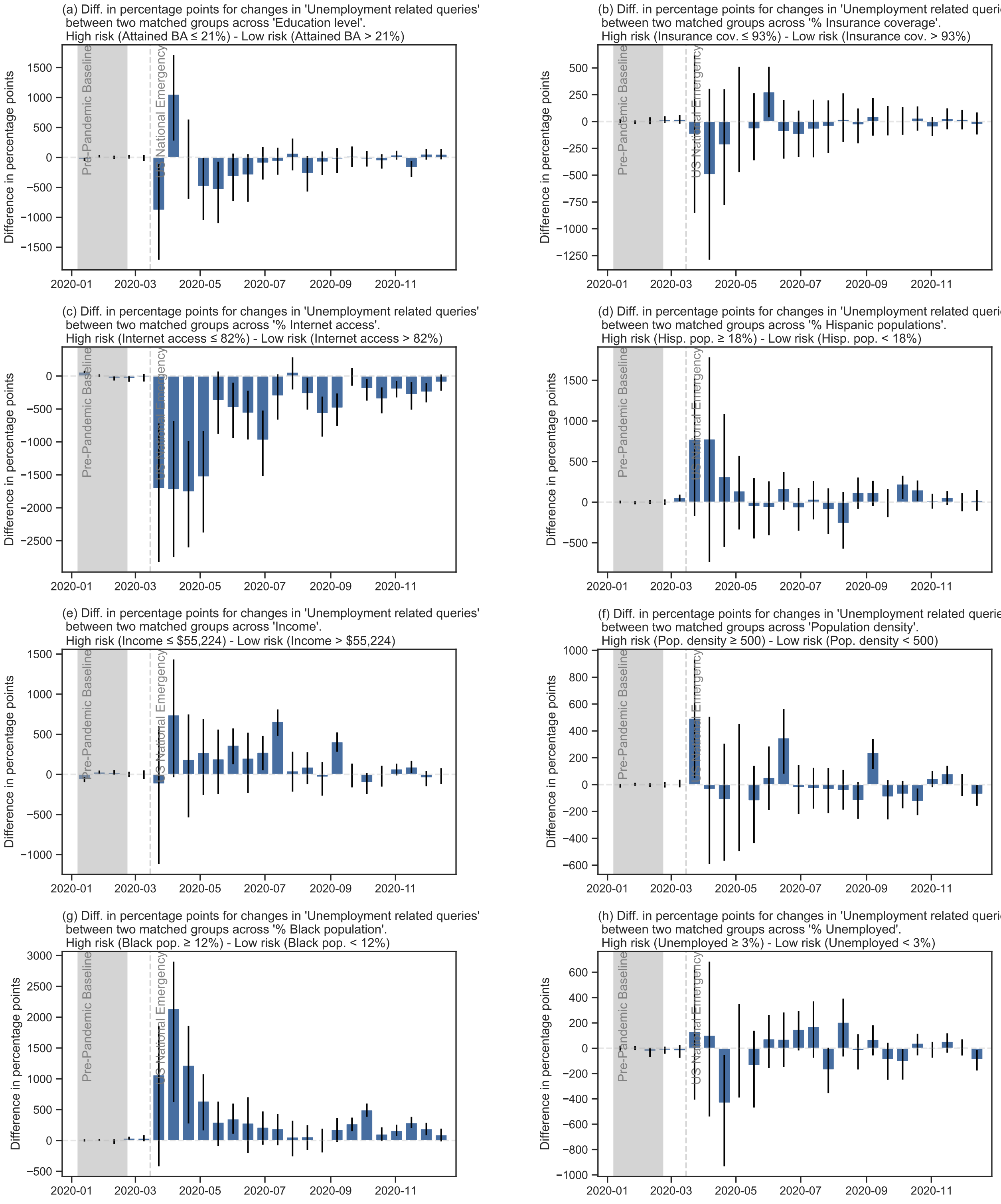

Supplementary Figure 16. Differences in percentage points for changes in 'Unemployment related queries' between two matched groups across eight SDoH factors. Data are presented as mean values, and error bars indicate 95% confidence intervals (1,603 $\leq n \leq$12,575, see Supplementary Table 6 for sample sizes per SDoH factor). Both mean values andconfidence intervals are obtained through bootstrapping with 500 iterations.

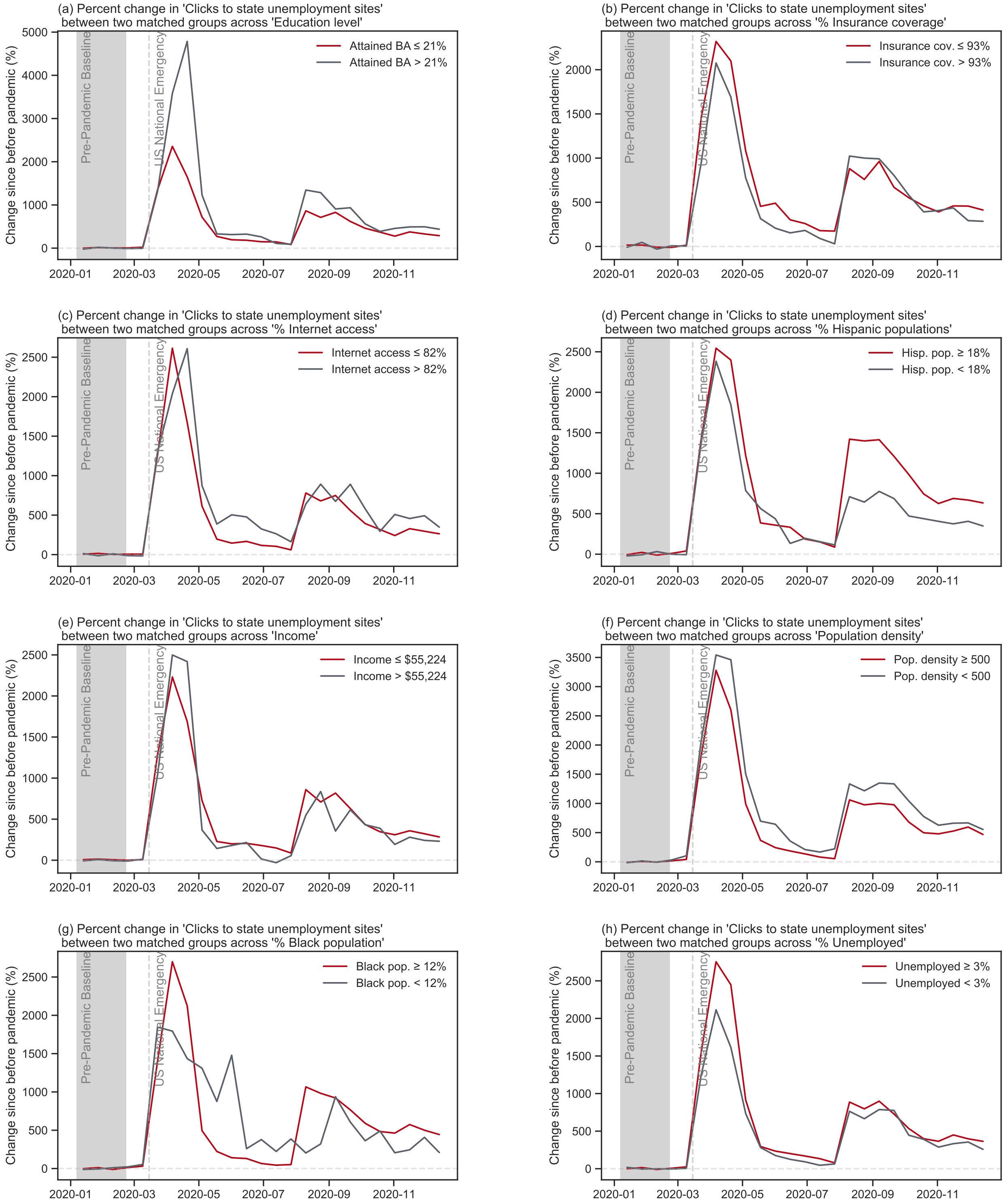

Supplementary Figure 17. Percent change in 'Clicks to state unemployment sites' between two matched groups across eight SDoHfactors.

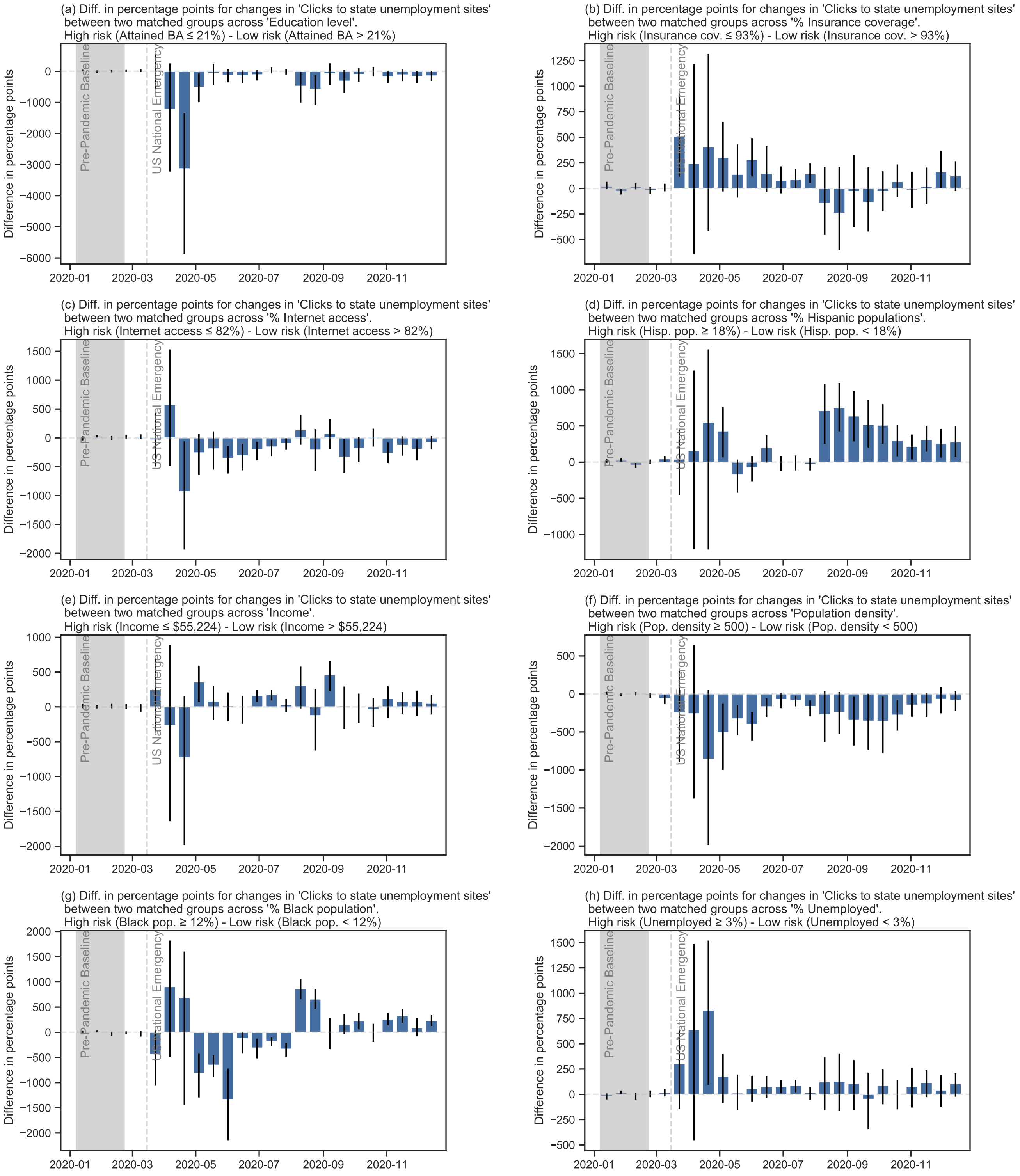

Supplementary Figure 18. Differences in percentage points for changes in 'Clicks to state unemployment sites' between two matched groups across eight SDoH factors. Data are presented as mean values, and error bars indicate 95% confidence intervals (1,603 $\leq n \leq$12,575, see Supplementary Table 6 for sample sizes per SDoH factor). Both mean values and confidence intervals are obtained through bootstrapping with 500 iterations.

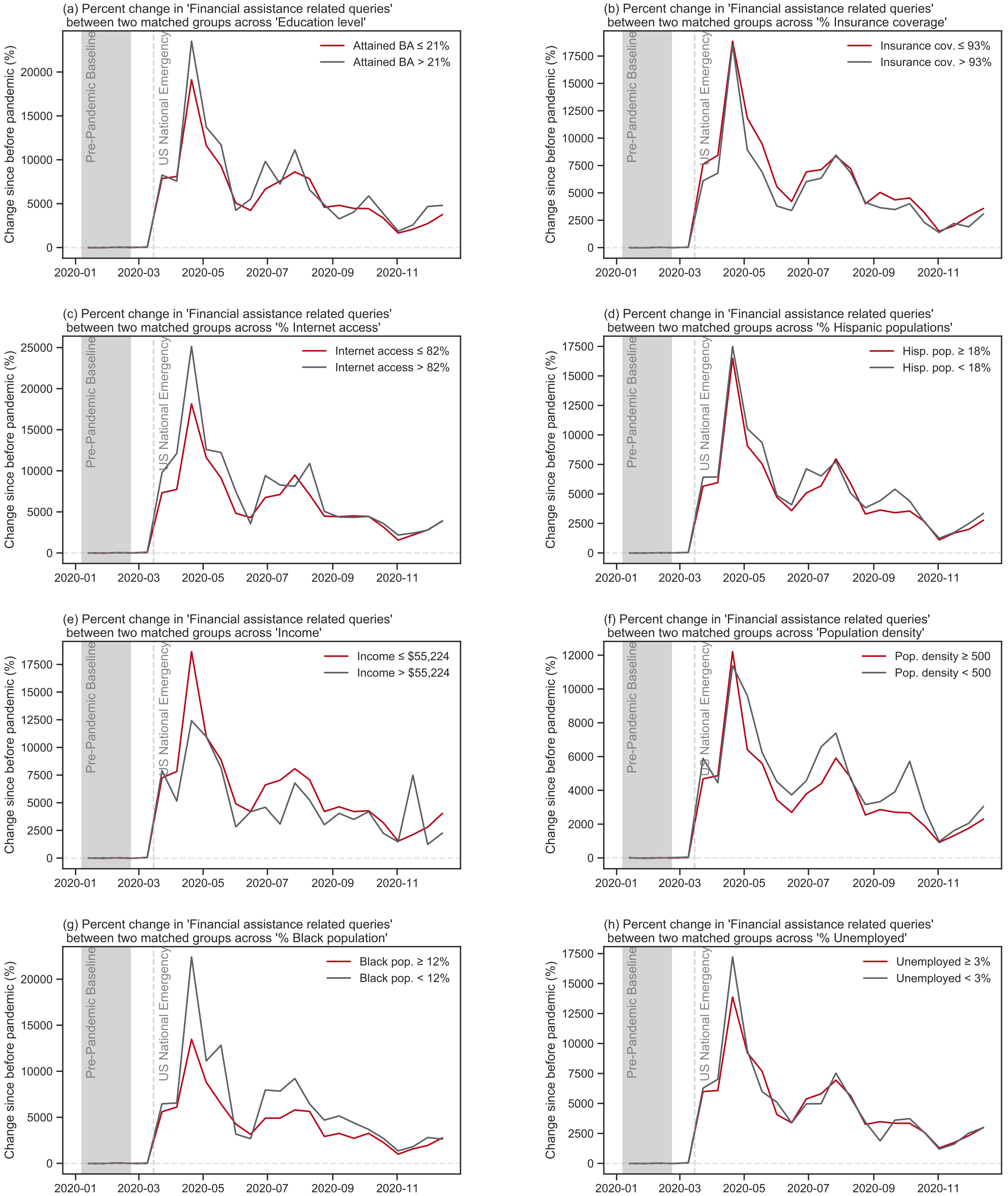

Supplementary Figure 19. Percent change in 'Financial assistance related queries' between two matched groups across eight SDoH factors.

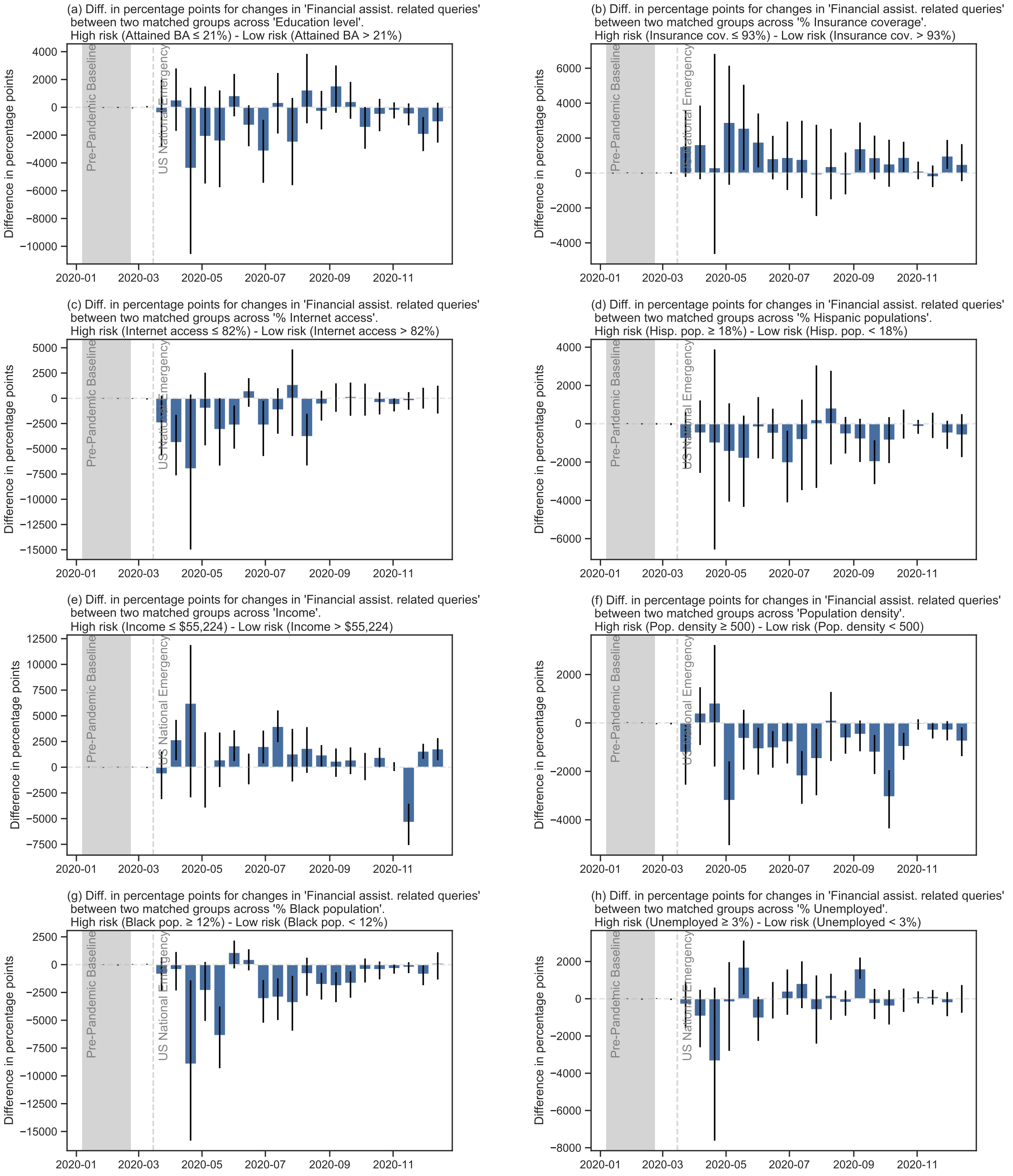

Supplementary Figure 20. Differences in percentage points for changes in 'Financial assistance related queries' between two matched groups across eight SDoH factors. Data are presented as mean values, and error bars indicate 95% confidence intervals (1,603 $\leq n \leq$12,575, see Supplementary Table 6 for sample sizes per SDoH factor). Both mean values and confidence intervals are obtained through bootstrapping with 500 iterations.

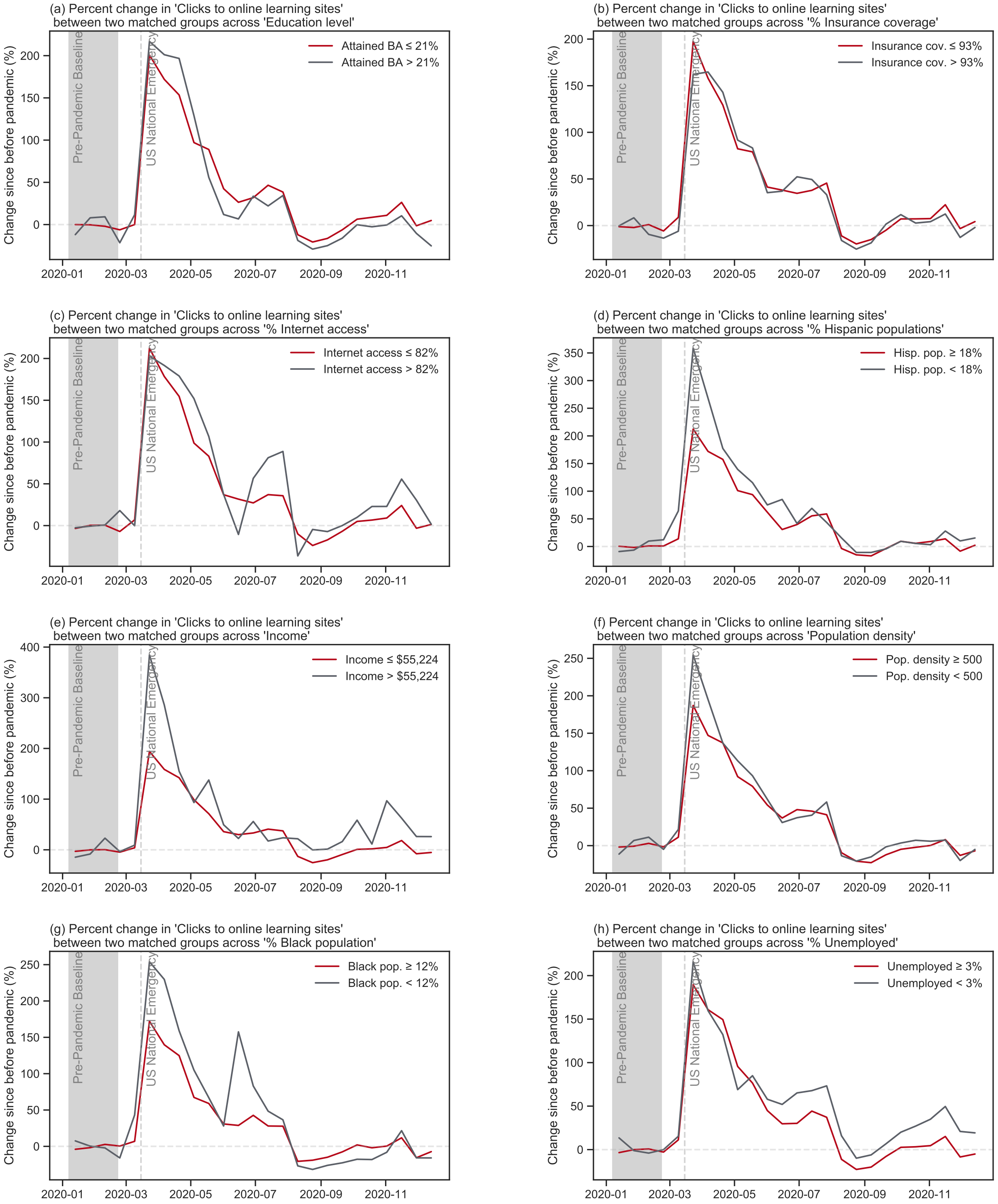

Supplementary Figure 21. Percent change in ‘Click to online learning sites’ between two matched groups across eight SDoH factors.

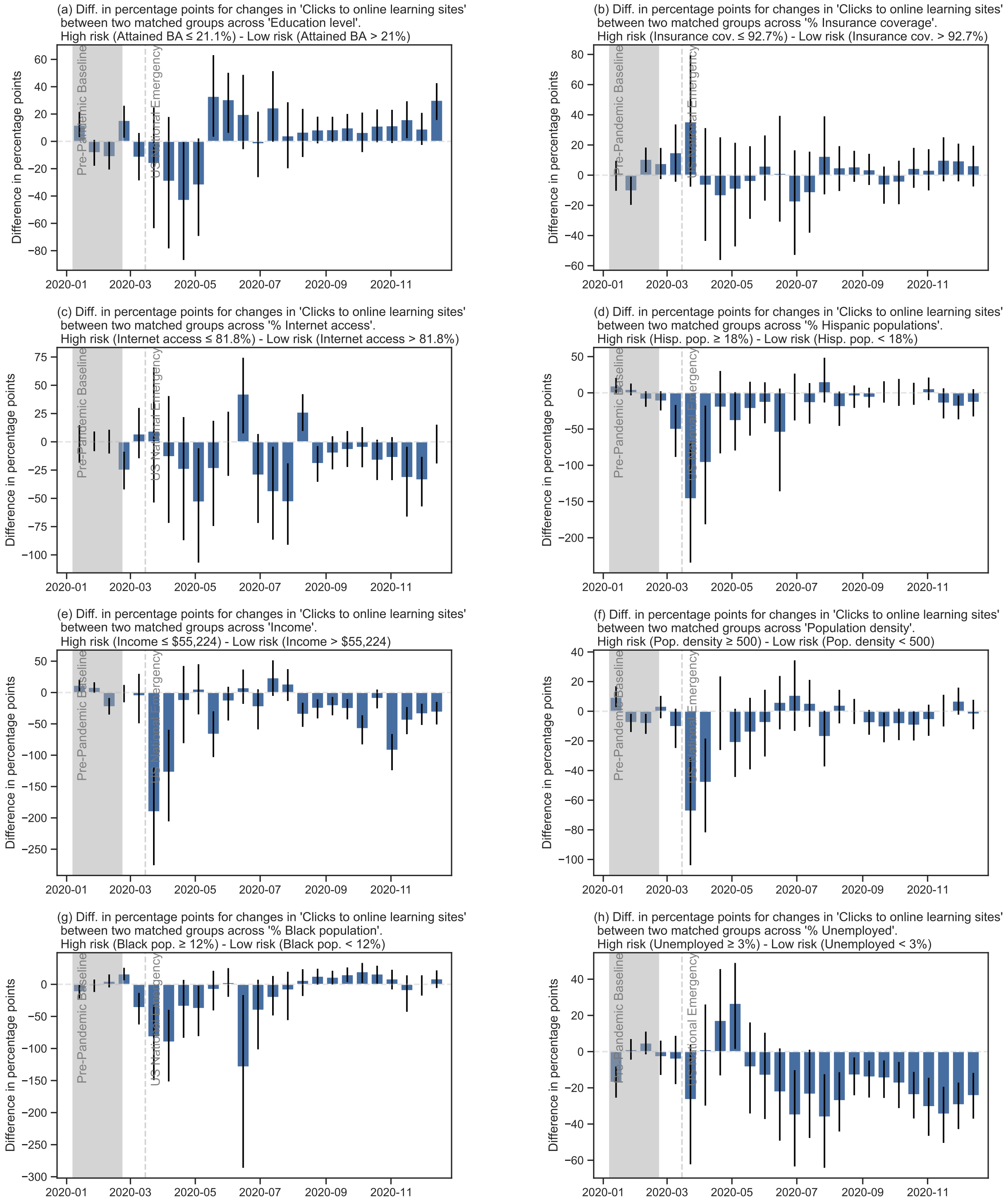

Supplementary Figure 22. Differences in percentage points for changes in 'Click to online learning sites' between two matched groups across eight SDoH factors. Data are presented as mean values, and error bars indicate 95% confidence intervals (1,603 $\leq n \leq$12,575, see Supplementary Table 6 for sample sizes per SDoH factor). Both mean values and confidence intervals are obtained through bootstrapping with 500 iterations.

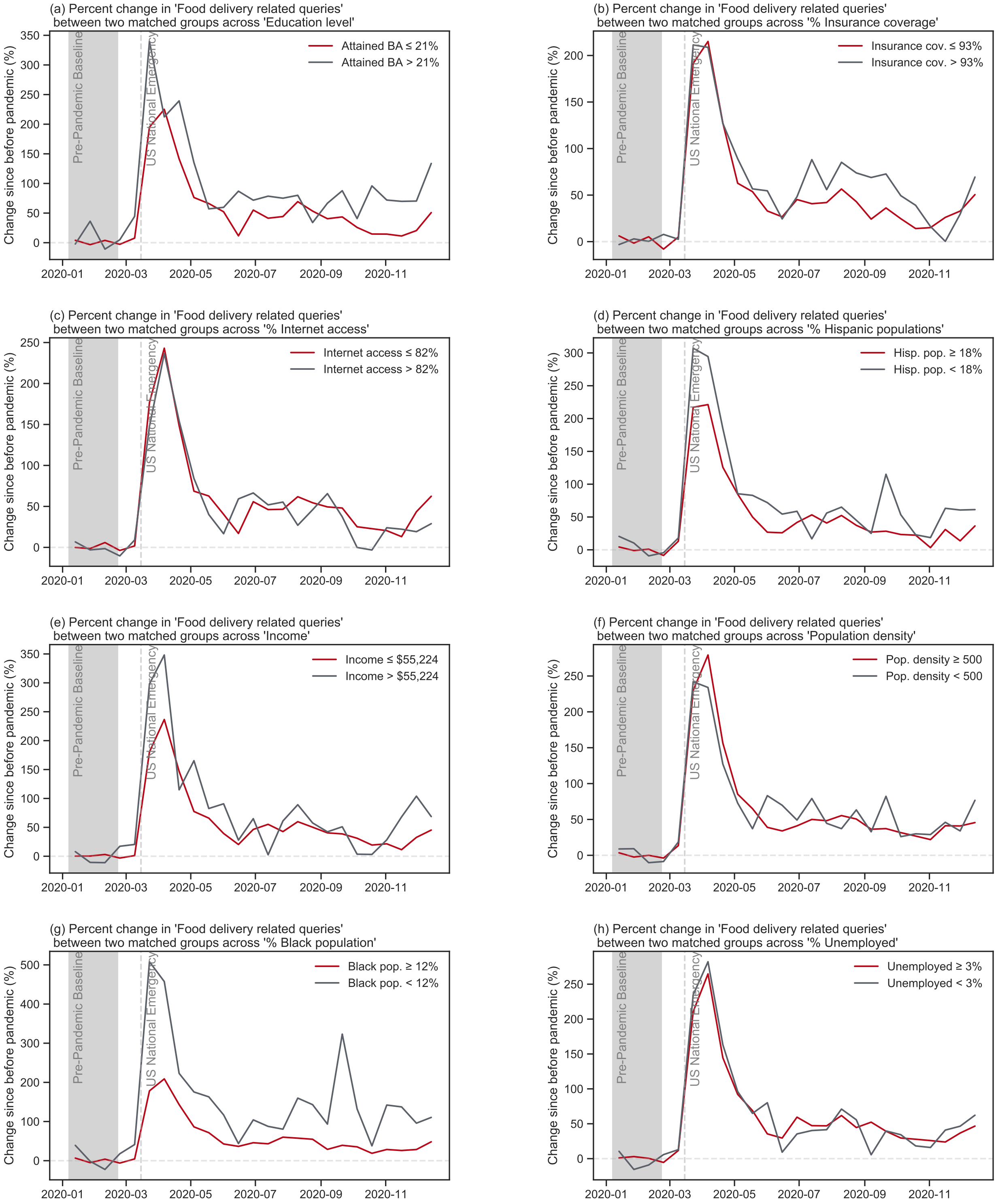

Supplementary Figure 23. Percent change in ‘Food delivery related queries’ between two matched groups across eight SDoH factors.

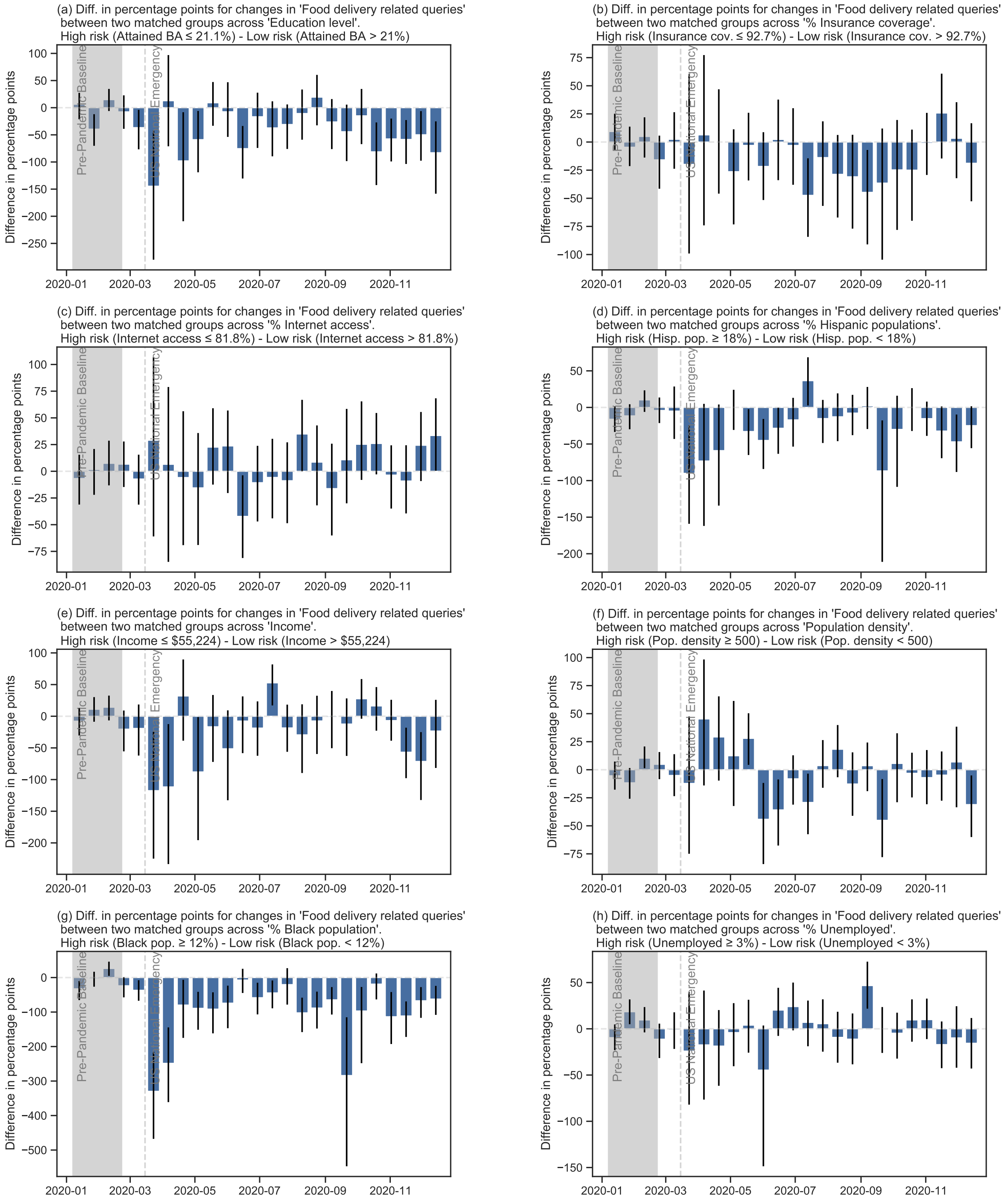

Supplemtary Figure 24. Differences in percentage points for changes in ‘Food delivery related queries’ between two matched groups across eight census variables. Data are presented as mean values, and error bars indicate 95% confidence intervals (1,603 $\leq n \leq$12,575, see Supplementary Table 6 for sample sizes per SDoH factor). Both mean values and confidence intervals are obtained through bootstrapping with 500 iterations.

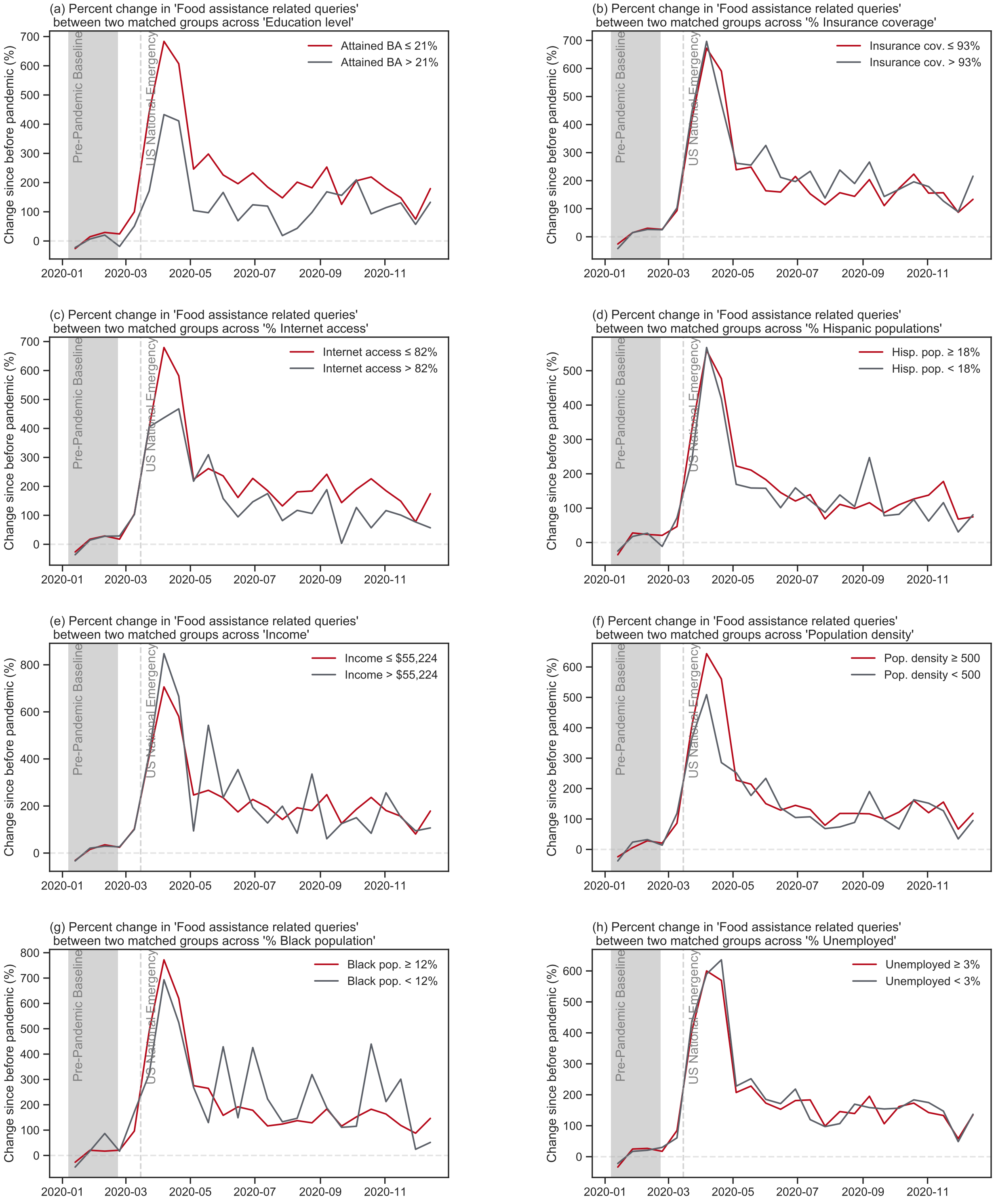

Supplementary Figure 25. Percent change in 'Food assistance related queries' between two matched groups across eight SDoH factors.

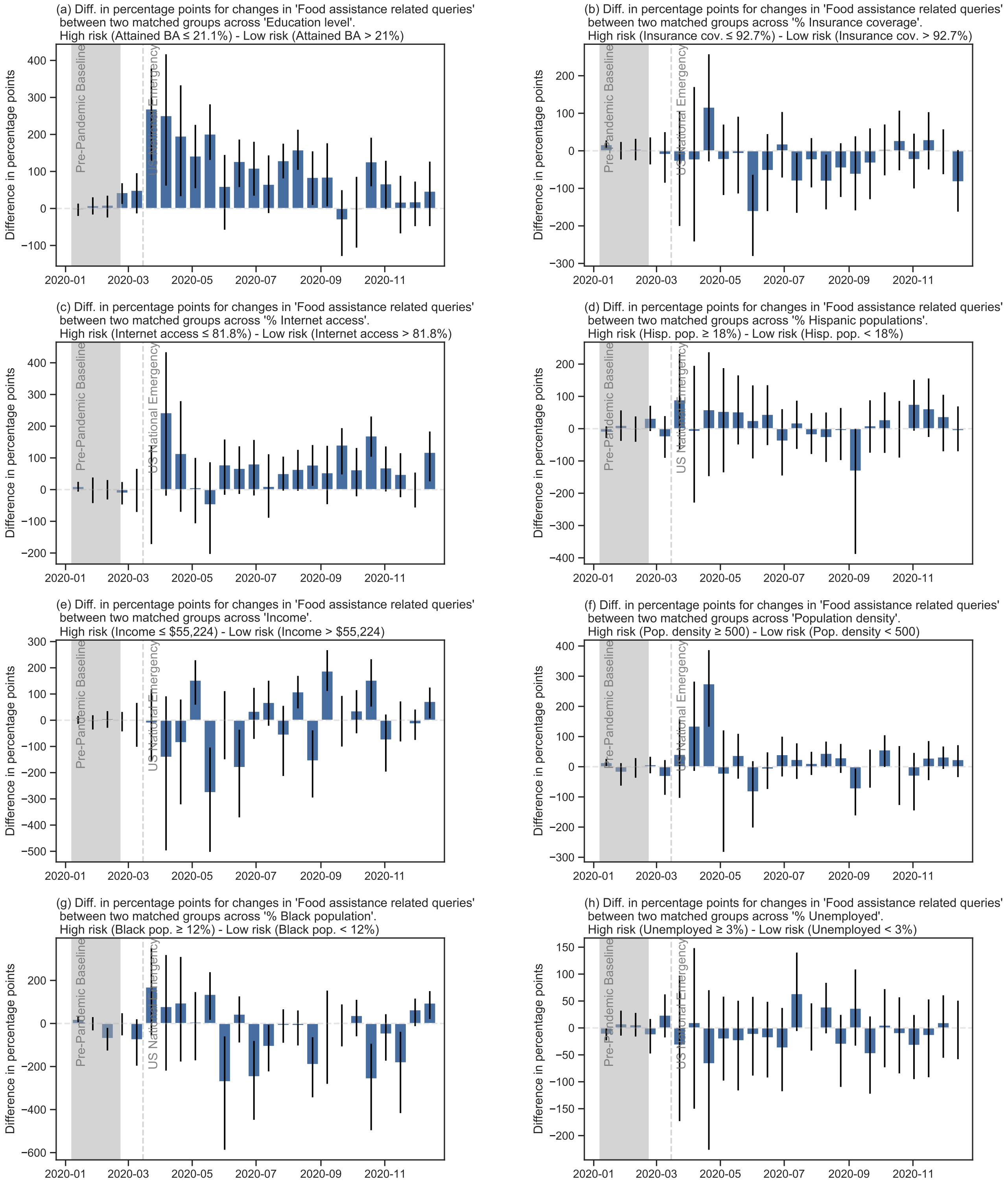

Supplementary Figure 26. Differences in percentage points for changes in 'Food assistance related queries' between two matched groups across eight SDoH factors. Data are presented as mean values, and error bars indicate 95% confidence intervals (1,603 $\leq n \leq$12,575, see Supplementary Table 6 for sample sizes per SDoH factor). Both mean values and confidence intervals are obtained through bootstrapping with 500 iterations.

Supplementary References

1. U.S. Census Bureau. American Community Survey 5-year estimates (2014-2018). https://censusreporter.org (Accessed 2020-06-08).

2. U.S. Census Bureau. State Area Measurements and Internal Point Coordinates. https://www.census.gov/geographies/reference-files/2010/geo/state-area.html (Accessed 2020-06-08).

3. Ratcliffe, M., Burd, C., Holder, K. & Fields, A. Defining rural at the US Census Bureau. *Am. community survey geography brief* **1** (2016).

4. Suh, J., Horvitz, E., White, R. W. & Althoff, T. Population-scale study of human needs during the covid-19 pandemic: Analysis and implications. In *Proceedings of the 14th ACM International Conference on Web Search and Data Mining*, 4–12 (2021).
