# Supplementary material for: Disparate impacts on online information access during the Covid-19 pandemic
Source: Nat Commun. 2022 Nov 19;13:7094. doi: 10.1038/s41467-022-34592-z (PMC9675823; doi:10.1038/s41467-022-34592-z)
Supplement: Supplementary file 2 — Reporting Summary [file 41467_2022_34592_MOESM2_ESM.pdf]

nature portfolio

Corresponding author(s): Tim Althoff

Last updated by author(s): Oct 7, 2022

Reporting Summary

Nature Portfolio wishes to improve the reproducibility of the work that we publish. This form provides structure for consistency and transparency in reporting. For further information on Nature Portfolio policies, see our Editorial Policies and the Editorial Policy Checklist.

Statistics

For all statistical analyses, confirm that the following items are present in the figure legend, table legend, main text, or Methods section.

n/a	Confirmed	
☐	☒	The exact sample size (*n*) for each experimental group/condition, given as a discrete number and unit of measurement
☐	☒	A statement on whether measurements were taken from distinct samples or whether the same sample was measured repeatedly
☒	☐	The statistical test(s) used AND whether they are one- or two-sided<br>*Only common tests should be described solely by name; describe more complex techniques in the Methods section.*
☐	☒	A description of all covariates tested
☒	☐	A description of any assumptions or corrections, such as tests of normality and adjustment for multiple comparisons
☐	☒	A full description of the statistical parameters including central tendency (e.g. means) or other basic estimates (e.g. regression coefficient) AND variation (e.g. standard deviation) or associated estimates of uncertainty (e.g. confidence intervals)
☒	☐	For null hypothesis testing, the test statistic (e.g. *F*, *t*, *r*) with confidence intervals, effect sizes, degrees of freedom and *P* value noted<br>*Give P values as exact values whenever suitable.*
☒	☐	For Bayesian analysis, information on the choice of priors and Markov chain Monte Carlo settings
☒	☐	For hierarchical and complex designs, identification of the appropriate level for tests and full reporting of outcomes
☐	☒	Estimates of effect sizes (e.g. Cohen's *d*, Pearson's *r*), indicating how they were calculated

Our web collection on statistics for biologists contains articles on many of the points above.

Software and code

Policy information about availability of computer code

Data collection	Raw data was collected by proprietary code through Microsoft Bing platform. Study data was extracted from Bing search logs stored on Microsoft's internal database and services and processed using its proprietary query language.
Data analysis	Data analysis was conducted in Python (v3.9.6) using standard data analysis libraries such as numpy (v1.20.3), scipy (v1.6.2), and pandas (v1.3.1). Visualization was produced using seaborn (v0.11.1). Matching was done using MatchIt (v4.2.0) in R.<br>The code supporting this study is retained indefinitely for scientific and academic purposes. The code is not publicly available due to employee privacy and other legal restrictions. The code is available from the authors on reasonable request and with permission from Microsoft Corporation.

For manuscripts utilizing custom algorithms or software that are central to the research but not yet described in published literature, software must be made available to editors and reviewers. We strongly encourage code deposition in a community repository (e.g. GitHub). See the Nature Portfolio guidelines for submitting code & software for further information.

Data

Policy information about availability of data

All manuscripts must include a data availability statement. This statement should provide the following information, where applicable:

- Accession codes, unique identifiers, or web links for publicly available datasets
- A description of any restrictions on data availability
- For clinical datasets or third party data, please ensure that the statement adheres to our policy

Raw US census data are publicly available through the Census Reporter API (https://censusreporter.org/). Geographical area measurements are available through the US Census Bureau (https://www.census.gov/geographies/reference-files/2010/geo/state-area.html). Seasonally adjusted US unemployment claims data for

2020 is available through the US Department of Labor (https://oui.doleta.gov/unemploy/claims.asp). The Bing search logs are not publicly available. An anonymized version of the data supporting this study is retained indefinitely for scientific and academic purposes. The data are not publicly available due to privacy and legal restrictions. The data are available from the authors on reasonable request and with permission from Microsoft Corporation.

Field-specific reporting

Please select the one below that is the best fit for your research. If you are not sure, read the appropriate sections before making your selection.

☐ Life sciences ☒ Behavioural & social sciences ☐ Ecological, evolutionary & environmental sciences

For a reference copy of the document with all sections, see nature.com/documents/nr-reporting-summary-flat.pdf

Behavioural & social sciences study design

All studies must disclose on these points even when the disclosure is negative.

Study description	We conduct a longitudinal before-after observational study with matched groups to answer noncausal questions of the form: How did the changes in search behaviors during the pandemic differ across matched groups delineated by a single socioeconomic and environmental factor? We analyze 57 billion de-identified search interactions in the United States from years 2019 and 2020 from Microsoft's Bing search engine to measure the changes in search behaviors for health, unemployment education, and food access during the pandemic for US ZIP codes. Based on the census data on each of the ZIP codes, we use 1-to-1 matching with replacement to control for any potential confounders. We finally compute the differences between these matched groups in their changes in search behaviors.
Research sample	Study data is 57 billion de-identified search interactions from Bing users in the United States from years 2019 and 2020 from Microsoft's Bing search engine. We have no other requirements for inclusion beyond the search interactions being from within the US based on our research target being the US population. Search engine users are not a representative sample of the US population.
Sampling strategy	No sampling is performed in obtaining search interactions.
Data collection	De-identified study data was extracted from Bing search logs stored on Microsoft's internal database and processed using its proprietary query language. Each search interaction includes the search query string, URLs of all subsequent clicks from the search result page, timestamp, and ZIP code from reverse IP lookup. US census data was obtained through the Census Reporter API (https://censusreporter.org/). Geographical area measurements were obtained through the US Census Bureau (https://www.census.gov/geographies/reference-files/2010/geo/state-area.html). Seasonally adjusted US unemployment claims data for 2020 was obtained through the US Department of Labor (https://oui.doleta.gov/unemploy/claims.asp). Data collection was independent of the experimental conditions or the hypotheses. No third-party was present during the data collection.
Timing	2019-12-20 to 2021-01-03
Data exclusions	We exclude 11,517 out of 36,667 ZIP codes because either (1) we have less than 100 queries per month from that ZIP code or (2) we do not have a corresponding census data we need for analysis.
Non-participation	No users are dropped out from aggregation.
Randomization	There was no randomization of ZIP codes (the smallest unit in our analysis). ZIP codes were allocated to treatment or control groups based on census data for those ZIP codes. We use a value close to the median to split the population into two groups for median household income ($55,224), % unemployed (3.0%), % with insurance (92.7%), % with internet access (81.8%), and % with Bachelor's degree or higher (21.1%) because the mean and median of those factors across the ZIP codes are similar. In other cases, the distribution across the ZIP codes are highly skewed. For race/ethnicity, we use the rounded percentage of the national population for that race/ethnicity (12% for Black and 18% for Hispanic populations). For population density, we follow previous practices of urban-rural classification (500 people per square mile). We control for potential confounders using 1-to-1 matching with replacement on ZIP codes across the two groups.

Reporting for specific materials, systems and methods

We require information from authors about some types of materials, experimental systems and methods used in many studies. Here, indicate whether each material, system or method listed is relevant to your study. If you are not sure if a list item applies to your research, read the appropriate section before selecting a response.

Materials & experimental systems

n/a	Involved in the study
☒	☐ Antibodies
☒	☐ Eukaryotic cell lines
☒	☐ Palaeontology and archaeology
☒	☐ Animals and other organisms
☐	☒ Human research participants
☒	☐ Clinical data
☒	☐ Dual use research of concern

Methods

n/a	Involved in the study
☒	☐ ChIP-seq
☒	☐ Flow cytometry
☒	☐ MRI-based neuroimaging

Human research participants

Policy information about studies involving human research participants

Population characteristics	Study data is de-identified search interactions from Bing users in the United States from years 2019 and 2020 from Microsoft's Bing search engine. We have no other requirements for inclusion beyond the search interactions being from within the US based on our research target being the US population. The data came from the user population in 36,667 US ZIP codes. We did not collect any population-specific information other than the ZIP code, and therefore, we cannot characterize the population further.
Recruitment	N/A - There was no recruitment
Ethics oversight	Microsoft Research Institutional Review Board

Note that full information on the approval of the study protocol must also be provided in the manuscript.
